# Epigenetics of Genes Preferentially Expressed in Dissimilar Cell Populations: Myoblasts and Cerebellum

**DOI:** 10.3390/epigenomes8010004

**Published:** 2024-01-26

**Authors:** Melanie Ehrlich, Kenneth C. Ehrlich, Michelle Lacey, Carl Baribault, Sagnik Sen, Pierre-Olivier Estève, Sriharsa Pradhan

**Affiliations:** 1 Tulane Cancer Center, Hayward Human Genetics Center, Center for Bioinformatics and Genomics, Tulane University Health Sciences Center, New Orleans, LA 70112, USA; 2 Center for Bioinformatics and Genomics, Tulane University Health Sciences Center, New Orleans, LA 70112, USA; kehrlich@tulane.edu; 3 Department of Mathematics, Tulane University, New Orleans, LA 70118, USA; mlacey1@tulane.edu; 4 Information Technology, Tulane University, New Orleans, LA 70118, USA; cbaribau@tulane.edu; 5 Genome Biology Division, New England Biolabs, Ipswich, MA 01938, USA; ssen@neb.com (S.S.); pesteve@neb.com (P.-O.E.); pradhan@neb.com (S.P.)

**Keywords:** myoblasts, cerebellum, epigenetics, WGBS, EM-seq, *ZNF556*, *CHD15*, *TRIM72*, *DOK7*, miR-486

## Abstract

While studying myoblast methylomes and transcriptomes, we found that *CDH15* had a remarkable preference for expression in both myoblasts and cerebellum. To understand how widespread such a relationship was and its epigenetic and biological correlates, we systematically looked for genes with similar transcription profiles and analyzed their DNA methylation and chromatin state and accessibility profiles in many different cell populations. Twenty genes were expressed preferentially in myoblasts and cerebellum (Myob/Cbl genes). Some shared DNA hypo- or hypermethylated regions in myoblasts and cerebellum. Particularly striking was *ZNF556*, whose promoter is hypomethylated in expressing cells but highly methylated in the many cell populations that do not express the gene. In reporter gene assays, we demonstrated that its promoter’s activity is methylation sensitive. The atypical epigenetics of *ZNF556* may have originated from its promoter’s hypomethylation and selective activation in sperm progenitors and oocytes. Five of the Myob/Cbl genes (*KCNJ12*, *ST8SIA5*, *ZIC1*, *VAX2*, and *EN2*) have much higher RNA levels in cerebellum than in myoblasts and displayed myoblast-specific hypermethylation upstream and/or downstream of their promoters that may downmodulate expression. Differential DNA methylation was associated with alternative promoter usage for Myob/Cbl genes *MCF2L*, *DOK7*, *CNPY1*, and *ANK1*. Myob/Cbl genes *PAX3*, *LBX1*, *ZNF556*, *ZIC1*, *EN2*, and *VAX2* encode sequence-specific transcription factors, which likely help drive the myoblast and cerebellum specificity of other Myob/Cbl genes. This study extends our understanding of epigenetic/transcription associations related to differentiation and may help elucidate relationships between epigenetic signatures and muscular dystrophies or cerebellar-linked neuropathologies.

## 1. Introduction

Skeletal muscle (SkM), the largest tissue in human body, has extraordinary regenerative ability and morphological plasticity in response to internal changes and external challenges [1,2]. SkM muscle progenitor cells play a central role in SkM formation and repair. Postnatally, there is a special mechanism of muscle repair involving the activation of muscle satellite cells, SkM stem cells that are usually dormant and lodged under the sarcolemma (the specialized outer membrane of SkM fibers) [3]. In response to SkM damage, satellite cells become activated to form myoblasts (SkM progenitor cells), which then differentiate and fuse with damaged myofibers. The muscle cells (myocytes) in myofibers contain hundreds or even thousands of nuclei.

The complexity of SkM formation and repair and the sensitivity of SkM to physiological and clinical changes makes the study of its epigenetics, including differentially methylated DNA regions (DMRs), of special interest. Because myocytes cannot divide, increases in muscle mass, e.g., due to exercise, rely on hypertrophy to enlarge the fibers radially and longitudinally [4]. Hypertrophy depends on both transcriptional changes in myofiber nuclei and activation of satellite cells [3,5]. Muscle injury can affect DNA methylation profiles of satellite cells [6]. During activation of satellite cells, there are further changes in DNA methylation and in chromatin [6,7]. There is overrepresentation of age-related SkM DMRs in SkM enhancer chromatin and regions around transcription start sites (TSS).

Epigenetics is also implicated in satellite cell, myoblast, and muscle fiber heterogeneity [8,9,10] and in memory effects for strenuous muscle use and, possibly, for muscle disuse [5,6,11,12]. There are differences in expression profiles and epigenetics for SkM muscle fiber subtypes (e.g., fast or slow), which can interconvert [10]. Muscle memory may be partly due to DNA methylation changes associated with SkM conditioning involving a bout of certain types of exercise followed by an interval of inactivity and a subsequent reinitiation of intense activity [5].

During a recent analysis of genes associated with myoblast DMRs, we found that *ZNF556* (Zinc Finger Protein 556) has a strong preference for expression in both myoblasts and cerebellum, as described below. In a previous study of myoblast epigenetics using reduced representation bisulfite sequencing (RRBS) profiles, we identified *CDH15* (Cadherin 15), as a gene also displaying myoblast and cerebellum-specific expression [13]. Cerebellum is critical for motor coordination, cognition, and emotional processes [14]. It is quite distinct morphologically and functionally from other brain regions and is important in neuromuscular disease [15]. Its transcriptome is strikingly distinct from those of all the other brain regions [16].

In this study, we systematically explored myoblast/cerebellum transcription associations. We first identified twenty human genes that are preferentially expressed in myoblasts and cerebellum (Myob/Cbl genes). We then investigated transcriptional and epigenetic relationships for Myob/Cbl genes using our newly available whole-genome bisulfite sequencing (WGBS) or enzymatic methyl-seq (EM-seq) myoblast methylomes [17], a recent WGBS profile for cerebellum neurons from Loyfer et al. [18], and chromatin epigenomics databases [19]. Unlike RRBS, which covers only up to 5% of the CpGs [20], WGBS and EM-seq allow the quantitation of methylation at essentially all the CpGs in the genome. These transcriptomic/epigenomic analyses using data from diverse tissues and cell cultures for comparison to myoblasts and cerebellum elucidate differences and similarities in the regulation of genes that are preferentially expressed in myoblasts and cerebellum, two very different kinds of cell populations.

## 2. Results

### 2.1. Genome-Wide Search for Genes Preferentially Expressed in Both Myoblasts and Cerebellum

Given our above-mentioned preliminary findings of myoblast/cerebellum preferential expression of several genes [13,21], we determined the frequency of such preferences for dual expression in a large set of human protein-coding genes. We examined transcription data for 13,847 genes that were in both the GTEx tissue RNA-seq database (52 tissue types [16]) and an ENCODE RNA-seq database for cell cultures (six of the nine cell cultures not derived from cancers). We required that the genes display appreciable expression in at least one of the tissues and one of the cell cultures (TPM, transcripts per million, or FPKM, fragments per kilobase million, ≥1). This gene set had been first depleted of most noncoding RNA (ncRNA) genes. The six types of cell cultures were myoblasts, lung fibroblasts (NHLF), keratinocytes (NHEK), umbilical vein endothelial cells (HUVEC), a B-cell lymphoblastoid cell line (LCL, GM12878), and embryonic stem cells (ESC, H1). Preferential expression of a gene in myoblasts was defined as an expression ratio of 5 for myoblast FPKM to the average FPKM of the five heterologous cell cultures and a myoblast TPM ≥1. For cerebellum, preferentially expressed genes were defined as those with the cerebellum TPM vs. the average TPM of 10 other brain regions ≥5 and the cerebellum TPM vs. the average TPM of 41 non-brain tissues ≥5. The TPM in cerebellum also had to be ≥1.

We found that 422 genes were preferentially expressed in myoblasts (Myob genes; 3.0% of the 13,847 genes) and 239 genes were preferentially expressed in cerebellum (Cbl genes; 1.7% of the 13,847 genes; Tables S1 and S2). The strongest enrichment in functional or structural terms (DAVID analysis [22]) among the Myob genes was for muscle contraction genes (35 genes; *p* = 2 × 10^−35^). Among the Cbl genes, genes involved in neurogenesis were the most enriched (16 genes, *p* = 3 × 10^−7^). A comparison of preferential expression in each of the brain regions confirmed [16] that cerebellum is clearly the brain region with the largest number of preferentially expressed genes (Table S3). We then determined that ~5% (20) of the Myob genes were also preferentially expressed in cerebellum (Myob/Cbl genes; Table 1 and S2). Twelve of the twenty Myob/Cbl genes were assigned Gene Ontology (GO) terms related to the SkM and/or neural lineages (Table S4).

To test whether cell cultures other than myoblasts also share preferential expression with cerebellum, we first identified NHLF-, LCL-, HUVEC-, NHEK-, or ESC-preferentially expressed genes among the 13,847-gene dataset. Preferentially expressed genes were again defined as those with expression ratios of ≥5 for the FPKM of each of these cell cultures to the average FPKM of five of the heterologous cell cultures (including myoblasts) and an FPKM for the cell culture of interest ≥1. The overlap of the resulting five gene sets with the set of Cbl genes gave the following numbers of genes that were preferentially expressed in both cerebellum and ESC, LCL, NHEK, HUVEC, or NHLF: 68, 16, 15, 11, and 4, respectively (Table S5).

Of the genes preferentially expressed in both cerebellum and one of the six cell cultures, only the Myob/Cbl genes were significantly (*p* < 0.01) enriched in “homeobox” and/or “developmental protein” terms (Table S5; *PAX3*, *EN2*, *LBX1* and *VAX1* and *ZIC1*, *EN2*, *CHRD*, *PAX3*, *LBX1*, *VAX2*, respectively). In contrast, among all 422 genes preferentially expressed in myoblasts, “homeobox” had less enrichment (*p* = 4 × 10^−7^) than muscle contraction genes (*p* = 2 × 10^−35^) and was not significantly enriched among all the 239 genes preferentially expressed in cerebellum. Fifteen of the twenty Myob/Cbl genes were associated with Myob DMRs (Table 1). Twelve of the twenty were expressed at a much higher level in myoblasts than in SkM (Table S4). We examined the epigenetic/transcription relationships of these fifteen genes using WGBS methylomes and RoadMap or ENCODE chromatin state segmentation and DNase-seq profiles.

### 2.2. CDH15, Which Encodes a Myoblast/Cerebellum Cadherin, Has a Hypomethylated 5′ Region in Myoblasts and Cerebellum

**Figure 1:**
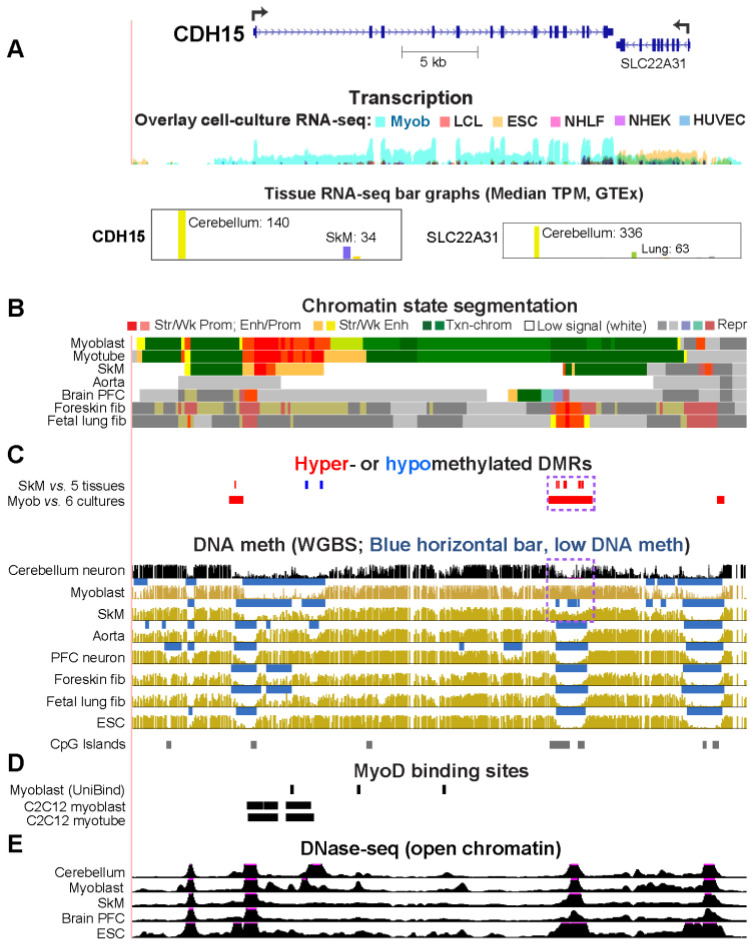
The 5′ end of *CDH15,* a cadherin gene expressed highly in both myoblasts and cerebellum, is hypomethylated in both cell populations. (**A**) The RefSeq isoform at chr16:89,230,125–89,270,723 (hg19). RNA-seq for cell cultures is depicted as the log transformed overlay signal and for 37 tissues as bar graphs with relative heights (linear scale) for median TPMs (GTEx; see Tables S1 and S2, for detailed data). Broken arrows depict the genes’ TSS and the direction of transcription. (**B**) Chromatin state segmentation (18-state; Roadmap Epigenomics Project) is based on key histone methylation and acetylation profiles. (**C**) DMRs and WGBS. In the DMR tracks, the purple dotted box indicates the cryptic promoter. For WGBS tracks, the blue bars are significantly low methylated regions (LMRs) relative to the whole genome for that sample. DMRs for SkM (psoas) vs. five other tissues (heart, aorta, lung, monocytes, and adipose tissue) and for myoblasts vs. six cell cultures (skin fibroblasts, lung fibroblasts, mammary epithelial cells, prostate epithelial cells, embryonic stem cells, and in vitro-generated adipocyte from mesenchymal stem cells) are shown. (**D**) CTCF and MyoD binding sites are from the UniBind ChIP-seq database or from human sequences homologous to C2C12 mouse DNA segments shown to bind MyoD upon ChIP-seq. (**E**) DNaseI hypersensitivity from ENCODE. Unless otherwise stated, all tracks are from the UCSC Genome Browser. Tracks are aligned except for the GTEx bar graphs. The dotted boxes in Panel **C** indicate the Myob-hyperm DMR described in the text. Abbreviations: Str, strong; Wk, weak; Prom, promoter; Enh, enhancer; Txn-chrom, chromatin with the H3K36me3 indicative of active transcription; Low signal, negligible signal for H3K27ac or me3, H3K4me1 or 3, H3K9me3, or H3K36me3; Repr, repressed; Myob, myoblasts; LCL, lymphoblastoid cell line (GM12848); ESC, human embryonic stem cells (H1); NHLF, lung fibroblasts; NHEK, normal human epidermal keratinocytes; HUVEC, human umbilical vein endothelial cells; SkM, skeletal muscle (psoas) except for GTEx data, where it indicates gastrocnemius SkM; PFC, pre-frontal cortex; fib, fibroblast; HMEC, human mammary epithelial cells.

*CDH15* (cadherin 15, M-cadherin) displayed the highest specificity for expression in cerebellum vs. other brain regions of all 20 Myob/Cbl genes (Table 1) as well as a high specificity for cerebellum vs. non-brain tissues (Table S2). We confirmed that *CDH15* was more highly expressed in myotubes than in myoblasts, as was previously reported [23]. Using RRBS methylomes and reporter gene assays, we previously found that *CDH15* has an intragenic cluster of Myob-hypermethylated CpGs [13]. It is part of a methylation-sensitive cryptic promoter highly active in myoblast host cells, but not in MCF7 (breast cancer cell line). With the recent availability of our myoblast WGBS/EM-seq-derived myoblast DMRs [17] and a cerebellum WGBS profile from Loyfer et al. [18], we re-examined the epigenetics of *CDH15*. It is not only preferentially but also strongly expressed in both cerebellum and myoblasts, and to a lesser extent in skeletal muscle (SkM; Figure 1A and Table 1). Its closest gene neighbor, *SLC22A31*, displays cerebellum- but not myoblast-specific transcription. The chromatin state profiles at *CDH15* reflect its expression profile (Figure 1B). Such data are not yet available for cerebellum. Myoblasts exhibited a low methylated region (LMR; a region with significantly lower methylation compared to the rest of the same genome [24]) extending downstream from the promoter region. Cerebellar neurons, myoblasts, and SkM also displayed lower methylation in this 5 kb region than did most other samples (Figure 1C). This region contained MyoD binding sites in myoblasts, as deduced from two ChIP-seq databases (Figure 1D). It also displayed DNaseI hypersensitivity (DNase-seq) peaks, which denote open/accessible chromatin, that were specific to either cerebellum or myoblasts (Figure 1E). The intragenic cryptic promoter, which overlaps two CpG islands (CGIs) and is specifically hypermethylated in myoblasts (Figure 1C, purple dotted box), was found to be larger than previously estimated from RRBS. This hypermethylation was absent in cerebellum. Cerebellum might not need to silence a cryptic promoter most active in myogenic cells. We also found that there was another Myob-hyperm DMR 0.7 kb upstream of the *CDH15* TSS, which had very low levels of methylation in cerebellar neurons. Therefore, only some of the presumably *cis*-acting differentially methylated regions in or adjacent to *CDH15* are shared between myoblasts and cerebellum in this gene, which is very highly and specifically expressed in both cell populations.

### 2.3. The Promoter Region of Transcription Factor-Encoding ZNF556 Is Hypomethylated Specifically in Tissues and Cells Expressing the Gene

**Figure 2:**
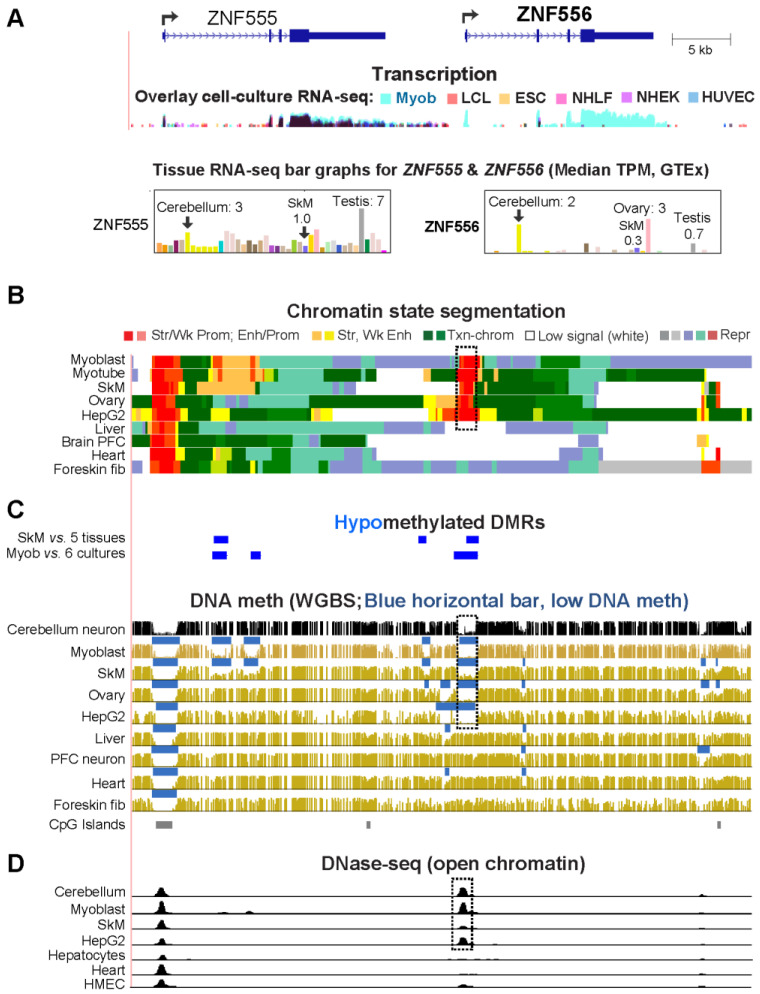
*ZNF556*, a KRAB-C2H2 zinc finger protein-encoding gene, exhibited a particularly strong association of myoblast DNA hypomethylation with gene expression. (**A**) The region shown (chr19:2,838,723–2,891,821) illustrates the RefSeq Select isoforms for *ZNF556* and *ZNF555* and transcription data, as in Figure 1. The dotted boxes in the epigenetic tracks of panels (**B**–**D**) indicate a hypomethylated region that overlaps the *ZNF556* promoter and a prominent DNase-seq peak seen in cells or tissues most strongly expressing *ZNF556*. HepG2, hepatocellular carcinoma cell line. Blue-green color for repressed chromatin in panel (**B**), a chromatin segment enriched in both H3K9me3 and H3K36me3, that is often seen in gene bodies of active or inactive *ZNF* family genes, especially at the 3′ end.

**Figure 3:**
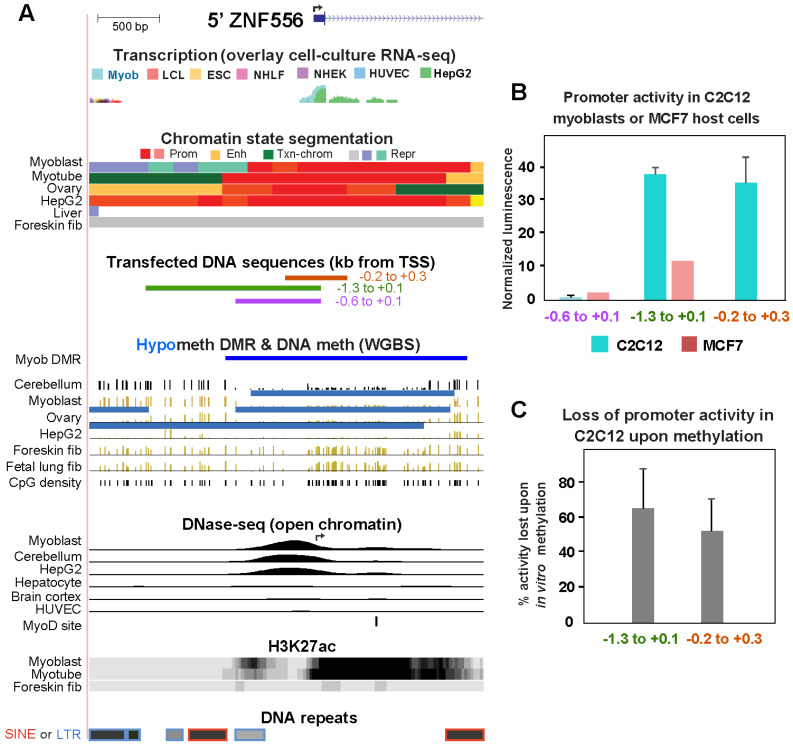
Hypermethylated sequences in the *ZNF556* promoter region have methylation-sensitive promoter activity in transfection assays. (**A**) The 5′ region of *ZNF556* (chr19:2,865,524–2,868,704) depicting the cloned sequences used in reporter gene assays and some of their epigenetic features. H3K27ac, H3K27 acetylation. The broken arrow in the DNase-seq tracks shows the position of the TSS, as in the top of the figure. The stand-alone dark blue horizontal bar depicts the hypomethylated DMR and the light blue horizonal bars above the WGBS profiles are the LMRs. DNA repeats are from the UCSC Genome Browser with the intensity of gray color indicating the extent of homology with consensus sequences; SINE (short interspersed repeats; Alu repeats), and LTR (long terminal repeat elements; ERV1 or ERVL families). (**B**) Normalized reporter gene activity for the indicated cloned DNA sequences transiently transfected as part of reporter constructs into C2C12 myoblasts or MCF7 cells. (**C**) Loss of promoter activity upon in vitro CpG methylation targeted to the cloned sequence.

Of the 20 Myob/Cbl genes, *ZNF556* (zinc finger protein 556) has the strongest specificity for expression in myoblasts (Table 1). It is a very little studied gene that encodes a protein which contains C2H2 zinc finger domains and a KRAB domain, as seen in many ZNF transcription factors (TFs). *ZNF556* is also specifically expressed in cerebellum, ovaries, HepG2 (liver cancer cell line), and testes (Figure 2A and Figure 3A, top). It has lower expression in SkM than in myoblasts, which suggests that its main function is in differentiation. Only *ZNF556*-expressing cell types and tissues displayed promoter chromatin and low DNA methylation both upstream and downstream of the gene’s TSS (Figure 2B,C and Figure 3A). In addition, only expressing samples had a prominent DNase-seq peak in this region, which was seen immediately upstream of the TSS (Figure 2D and Figure 3A).

The nearest neighbor to *ZNF556* is *ZNF555*, whose 3′ end is 7 kb upstream of the *ZNF556* TSS (Figure 2A). *ZNF555* encodes a TF [25] that, like ZNF556, is a member of the KRAB-ZNF family. *ZNF555* had a much broader tissue and cell culture expression profile than *ZNF556* although its RNA levels in myoblasts were about twice that of the average of five other cultures. Like *ZNF556*, *ZNF555* was expressed at high levels in testis relative to other tissues (TPM testis/average 36 other tissues = 4.3). The three other genes within the 150 kb neighborhood of *ZNF556* also encode zinc finger proteins, but none shows preferential expression in myoblasts. One does (*ZNF57*) does in testis (Figure S1). Myoblast-associated enhancer chromatin was missing within *ZNF556* or at intergenic regions but was present within the gene body of *ZNF555* overlapping Myob-hypom DMRs (Figure 2B). Therefore, the intragenic SkM-lineage associated *ZNF555* enhancer chromatin may help upregulate *ZNF556* expression in myoblasts and myotubes.

Because of the strong association of *ZNF556* expression with its promoter hypomethylation, we cloned several fragments from its 5′ end (Figure 3A) to test the effect of in vitro CpG methylation at the cloned sequences on promoter activity. Surprisingly, we found that DNA sequences from 0.6 kb upstream of the TSS to 0.1 kb downstream (Figure 3A, −0.6 to +0.1) had negligible promoter activity in C2C12 myoblasts despite their overlap with the myoblast-associated DNase-seq peak, the Myob-hypom DMR, and an apparent nucleosome-free region as seen in the myoblast histone H3 lysine-27 acetylation profile (H3K27ac; Figure 3A,B). However, moderately strong promoter activity was observed in C2C12 myoblasts when the cloned DNA sequences were extended upstream (−1.3 to +0.1 kb relative to the TSS) or slightly downstream (−0.2 to +0.3 kb instead of −0.6 to +0.1 kb). No activity or lower activity was observed in MCF7 cells with these constructs (Figure 3B). Therefore, the +0.1 to +0.3 and the −0.6 to −1.3 kb sequences may contribute to promoter function in vivo even though the latter are normally methylated in myoblasts at their low CpG-density DNA sequence (Figure 3A). When the two reporter constructs that contained promoter activity were CpG-methylated in vitro by M.SssI only at the cloned sequences, about half to 60% of the promoter activity was lost (Figure 3C). These findings indicate cooperative interactions to establish promoter activity involving sequences outside of DNase-seq (open chromatin) peaks interacting with DNA sequences in the DNase-seq peak region.

**Figure 4:**
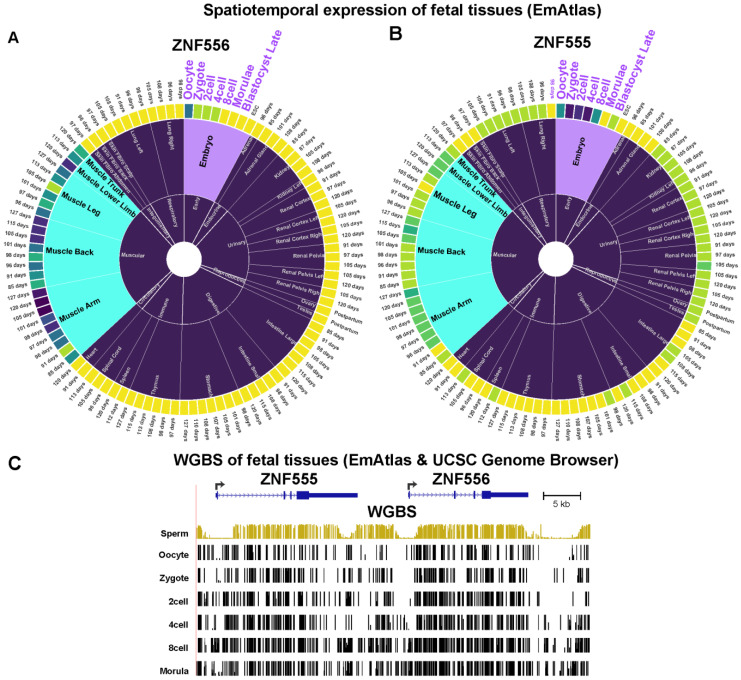
*ZNF555* and *ZNF556* RNAs are present in cells from early pre-implantation embryos, in which their genes display *ZNF556* promoter hypomethylation. (**A**) Relative levels of *ZNF556* and (**B**) *ZNF555* RNAs in early embryo and fetal tissues, adapted from the EmAtlas’ compiled data [29]. For clarity, the inner sectors for fetal skeletal muscle and early embryo are colored blue and purple, respectively. In the outer ring, yellow indicates no detectable expression, and the intensity of the other colors is a reflection of the relative scRNA-seq signal. The maximum nTPM for *ZNF556* was 32 in fetal arm muscle and for *ZNF555* was 38 in the zygote. (**C**) WGBS profiles from the EmAtlas and, for sperm, from the UCSC Genome Browser for the hg38 coordinates chr19:2,838,727–2,891,825.

Although the role of *ZNF556* in development is unclear, there are indications of its importance from additional aspects of its transcriptional and epigenetic specificity. First, its expression in human fetal cells is highest in SkM myocytes and satellite cells, as determined from single-cell RNA-seq (scRNA-seq) profiles (UCSC Genome Browser, hg38, [26]). Among various non-brain mid-gestation tissues, the expression of *ZNF556* (but not *ZNF555*) is much higher in muscle than in other examined tissues (Figure 4A). From our previous bulk RNA-seq data for myoblasts and myotubes [27], *ZNF556* RNA levels in myotubes were twice as high as in myoblasts. Importantly, in an extensive scRNA-seq analysis of many cell types and post-natal human tissues (not including cerebellum) [28], *ZNF556* RNA levels are highest in ovarian stromal cells, oocytes, and spermatogonia, as well as in skeletal myocyte, esophageal squamous epithelial cells, and plasmacytoid dendritic cells. Transcriptome analysis of early embryos as part of the EmAtlas collection [29] indicates the presence of *ZNF556* and *ZNF555* in zygotes, two-cell, and four-cell human embryos (Figure 4A,B). In addition, WGBS profiles of sperm, oocytes, zygotes, two-cell, four-cell, eight-cell and morula embryos [29,30] show a loss of promoter region hypomethylation at the eight-cell stage, when *ZNF556* RNA is no longer detectable (Figure 4C). However, much RNA found at the earliest stages in the pre-implantation embryo is carried over from the oocyte to the zygote.

### 2.4. Differential Methylation of the Extended Promoter Regions of TRIM72 and Its Intronic Gene, PYDC, Correlates with Differential Expression in Myoblasts and Cerebellum

**Figure 5:**
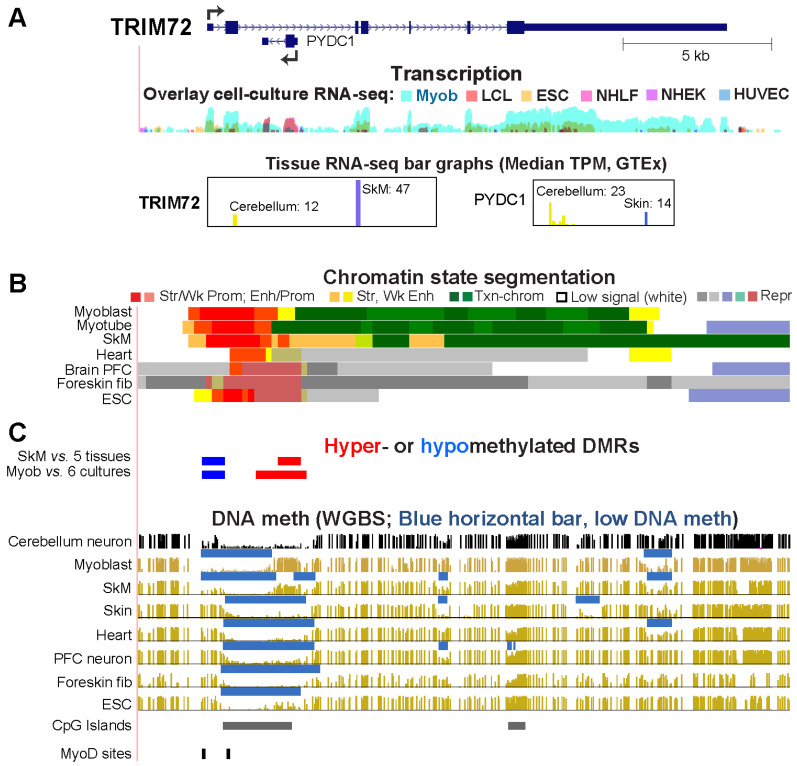
*TRIM72*, a myokine-encoding gene, harbors an antisense coding gene, *PYDC1*, that is hypermethylated and not expressed in myoblasts but hypomethylated in cerebellum. The only *TRIM72* and *PYDC1* RefSeq Curated isoforms are shown at chr16:31,223,125–31,244,961. Panels (**A**–**C**) are similar to panels in Figure 1.

*TRIM72* (tripartite motif containing 72) encodes a ubiquitin ligase that is involved in muscle regeneration, calcium homeostasis, excitation–contraction coupling, and mitochondrial autophagy [31]. It has a very high specificity for SkM and myoblasts as seen in RNA-seq profiles and chromatin epigenetic profiles (Figure 5A,B). TRIM72 is a myokine, a SkM secreted protein, with systemic effects on membrane repair and skin repair [32,33]. It is one of the unusual genes that harbors an antisense coding gene, *PYDC1*, within one of its introns. *PYDC1* encodes an inhibitor of NFκB [34]. Both *PYDC1* and *TRIM72* are specifically expressed in cerebellum but only *TRIM72* is also expressed in myoblasts and SkM. *PYDC1* is also strongly expressed in skin and suprabasal keratinocytes in skin [28] and in a primary keratinocyte cell culture (NHEK, Figure 5A, purple signal). Correlated with the transcription profiles, myoblasts and SkM displayed hypermethylation at the *PYDC1* promoter and throughout this 1 kb gene (Figure 5C). Although the lack of methylation in this region in *PYDC1*-expressing cerebellum and skin was consistent with expression in these tissues, there also was little or no methylation in tissues not expressing this gene. Therefore, the lack of methylation was not sufficient for expression. In contrast, only *TRIM72*-expressing samples (myoblasts, SkM, and to a lesser extent, cerebellum) displayed hypomethylation in the *TRIM72* promoter region. This DMR extends upstream of a constitutively unmethylated CGI whose 5′ end is at exon 2.

### 2.5. Alternate ANK1 Promoter Usage in Myoblasts vs. Cerebellum Is Associated with Differential Promoter Methylation

**Figure 6:**
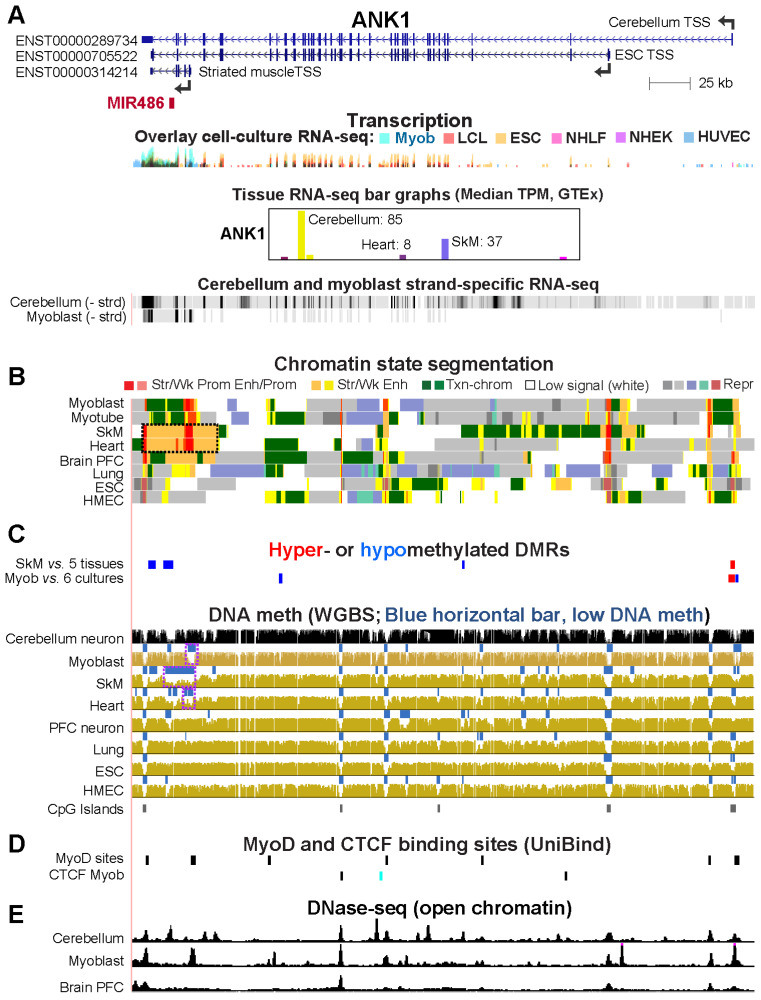
Hypomethylation at the myoblast/skeletal muscle/heart-associated promoter for ankyrin-encoding *ANK1*. Most of the many RefSeq Curated or Ensembl isoforms, including ENST00000265709 which has the most distal promoter (GTEx database), are not in the depicted coordinates, chr8: 41,508,221–41,660,626. (**A**–**E**) are similar to panels depicted in Figure 1 with the addition of CTCF binding sites from Unibind; blue, the CTCF site was seen in myoblasts but not in HMEC or ESC; black, the CTCF binding sites that were seen in myoblasts and ESC or HMEC. In addition to the ENCODE RNA-seq overlay profiles, panel (**A**) shows Roadmap or ENCODE strand-specific RNA-seq signal intensities. Dotted box in Panel (**B**), super-enhancer seen in SkM and the heart. Dotted boxes in panel C indicate the LMRs specific to myoblasts, SkM, and heart that overlap the alternate *ANK1* promoter for the smallest RNA isoform.

*ANK1* (ankyrin 1) codes for a protein that links integral membrane proteins to the cytoskeleton and is involved in cell motility, proliferation, and the maintenance of specialized membrane domains [34]. It is very highly expressed in erythroblasts and preferentially transcribed in cerebellum, SkM, and, to a lesser extent, heart (Figure 6A) [26,34]. It has many tissue-specific RNA isoforms (see Figure 6A for several of them) involving alternative promoter usage as well as alternative splicing. SkM [35], myoblasts, and heart transcribe predominantly one of the shortest isoforms (ENST00000314214) while cerebellum transcribes predominantly one of the longest isoforms (ENST00000289734, Figure 6A and GTEx isoform expression profiles [16]). Myoblasts express this gene at the highest levels compared to 17 other non-transformed cell cultures in the ENCODE database (Figure 6A). Some cell cultures unrelated to the muscle lineage express *ANK1* at moderate levels, e.g., ESC, bone marrow stem/stromal cells, and osteoblasts. However, these cell cultures do not use myoblasts’ proximal promoter, as indicated by 5′ cap analysis gene expression (CAGE) [36] of cell cultures. The 5′ ends of the large *ANK1* isoforms can be difficult to ascertain. For example, CAGE profiles show that ESC primarily use the promoter of the *ANK1* isoform ENST00000705522. GTEx indicates that cerebellum predominantly initiates transcription at a more distal promoter although this is not clear from RNA-seq profiles (Figure 6A). In the last intron of all the *ANK1* isoforms, *MIR486* is found. It encodes miR-486, a miRNA that is essential for myoblast proliferation and differentiation, normal myogenesis, and normal SkM and heart formation [37].

Tissue-specific differential *ANK1* promoter usage was reflected in the DNA methylation profiles and the chromatin state profiles (Figure 6B,C). Only SkM had a long LMR that overlapped the gene body of the main striated muscle lineage-associated short isoform, its promoter, and a super-enhancer [38] (>5 kb cluster of enhancer and promoter chromatin with strong H3K27ac; Figure 6B, dotted box). Heart also displays a super-enhancer in this region but, consistent with its much lower *ANK1* expression than in SkM, it has less extensive DNA hypomethylation (Figure 6A,B). At the striated muscle/myoblast promoter, myoblasts exhibited a smaller LMR than seen in SkM (Figure 6C). Under less stringent conditions of hypom DMR assignment (methylation difference of ≤−0.20 instead of our standard ≤ −0.35), a Myob-hypom DMR coincided with the myoblast LMR. *ANK1*-expressing cerebellum as well as tissues/cells that do not express *ANK1* were highly methylated in this region (Figure 6C). The cerebellum promoter for *ANK1* overlaps enhancer chromatin in SkM and heart and a CGI (Figure 6A,C). Importantly, most of this CGI overlapped a Myob/SkM-hyperm DMR, which has a Myob-hypom DMR upstream (Figure 6C). These results suggest that the Myob-hyperm DMR represses formation of the cerebellum promoter but also permits this region to be harnessed as an enhancer. Consistent with this interpretation, a strong DNase-seq peak and a MyoD site in myoblasts overlaps the far-upstream Myob-hypom DMR (Figure 6D,E).

### 2.6. Myoblast DNA Hypermethylation of PAX3 May Be Downmodulating Gene Expression by Suppressing Super-Enhancer Formation

**Figure 7:**
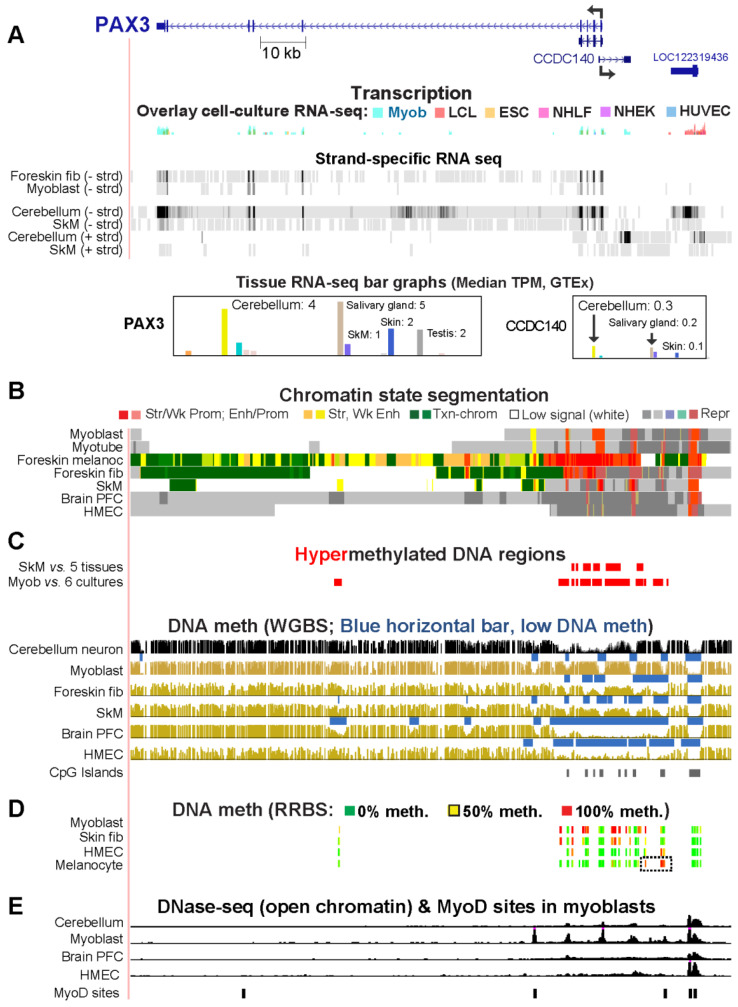
Hypermethylated DMRs upstream and downstream of the promoter region of the TF-encoding *PAX3* may downmodulate expression of this gene in both myoblasts and cerebellum. The RefSeq Select isoform is shown at chr2:223,058,677–223,192,231. (**A**–**C**) and (**E**) are similar to panels in Figure 6. (**D**) RRBS DNA methylation data from ENCODE with the indicated color coding; dotted box, the melanocyte-specific hypermethylated region.

*PAX3*, which encodes a developmental TF that plays critical roles in embryogenesis, including in neural development and myogenesis [34,39,40], is preferentially, but lowly, expressed in skin fibroblasts, myoblasts, and cerebellum (Figure 7A). Among postnatal somatic cells, it is much more highly expressed in melanocytes than in other cell types (Figure 7B and [28]). This is consistent with its role in melanocyte development and homeostasis [41]. Myoblasts exhibit hypermethylation at the 5′ end of the gene and upstream through the adjacent *CCDC140* ncRNA gene, as was seen previously in RRBS profiles [42]. A cluster of Myob-hypermeth DMRs, which were deduced from WGBS and EM-seq, extended from +0.4 to +9.7 kb and −0.5 to −14.6 kb relative to the *PAX3* TSS. RRBS data indicated that this region was mostly unmethylated in melanocytes (with the exception of a far-upstream subregion (Figure 7D, dotted box)) and in 15 primary cell cultures and 13 tissues that show little or no expression of this gene (Figure 7C,D; [43]). This long unmethylated region in *PAX3*-nonexpressing cells/tissues overlaid repressed chromatin. In cerebellum, foreskin fibroblasts, and SkM, as well as in myoblasts, all of which express this gene (Figure 7A; [28]), there was a cluster of highly methylated subregions in this region (Figure 7C). The Myob-hyperm DMRs and high-methylation subregions in cerebellum were interspersed with methylation valleys that correspond to tissue/cell-specific DNase-seq peaks in myoblasts and cerebellum and occasionally MyoD binding sites in myoblasts (Figure 7C,E). These findings suggest that a major function of the hypermethylation in myoblasts and cerebellum is to downmodulate expression of this gene in myoblasts and cerebellum by helping to prevent the formation of the 5′ super-enhancer seen in highly expressing melanocytes. In addition, DNA hypermethylation may suppress expression of the very weakly expressed *CCDC140* ncRNA gene which shares a bidirectional promoter with *PAX3*. Because of the two genes’ strikingly similar transcription profiles, this very little studied ncRNA gene, which overlaps Myob-hyperm DMRs, may help regulate *PAX3* expression.

### 2.7. Other Genes Preferentially Expressed in Myoblasts and Cerebellum Profiles Display Myoblast- or Cerebellum-Associated Hypomethylation and Overlapping Transcription Factor Binding Sites

Ten additional Myob/Cbl genes, which are associated with Myob DMRs were examined for epigenetic/transcriptional associations (Figures S2–S10). Seven of these (*ZIC1*, *CHRD*, *KCNJ12*, *VAX2*, and *ST8SIA5*, and the gene neighbors *EN2* and *CNPY1*) are expressed much more highly in cerebellum than in myoblasts (Table 1). All but one of the seven genes, *CHRD* (chordin), had Myob-hyperm DMRs that were missing in cerebellum. These DMRs were near promoter regions, which were sometimes hypomethylated specifically in cerebellum. *CHRD* had its only Myob-DMR as a hypomethylated DMR overlapping Myob-associated enhancer chromatin but had multiple intragenic regions of hypomethylation in cerebellum (Figure S3). The methylomes for *LBX1* (ladybird homeobox 1) indicate that its Myob-hyperm DMRs are likely downregulating its expression in myoblasts (Figure S8). However, the lack of chromatin state profiles for cerebellum at these regions, that are largely unmethylated regions in cerebellum (*LBX1* expressing) and most tissues (*LBX1* silent), makes interpreting their DNA methylation/transcription relationships difficult (Figure S8).

*MCF2L* displayed associations between differential DNA methylation at alternative promoters and alternate promoter usage in myoblasts vs. cerebellum (Figure S9). *DOK7* (docking protein 7) displays a Myob-hyperm DMR as well as DNA hypermethylation in cerebellum immediately downstream of an alternative intragenic promoter (Figure S10). The canonical upstream promoter, rather than the downstream one, is used preferentially in myoblasts and cerebellum and some other expressing cell cultures and tissues (Figure S10 and GTEx isoform profiles [16]). In contrast, mammary epithelial cells (HMEC, Figure S10) and B cells use the downstream promoter and are hypomethylated in this TSS-downstream region. *DOK7* is, therefore, an example of Myob/Cbl gene with regions displaying similar cell/tissue-specific DNA methylation and alternative promoter usage in myoblasts and cerebellum.

We examined the six Myob/Cbl genes *(ZNF556*, *TRIM72*, *ANK1*, *NRXN2*, *LBX1*, and *MCF2L*) that had Myob-hypom DMRs to ascertain whether cerebellum also had an overlapping region of unusually low DNA methylation. This was the case for *ZNF556*, *ANK1*, *TRIM72*, and *MCF2L*. Within three of these Myob-hypom DMRs, we found predicted TF binding sites (TFBS) in the JASPAR database for TFs that are encoded by Myob/Cbl genes (*LBX1*, *EN2*, and *ZIC1*) (Figure S11), but none of the six TFs encoded by Myob/Cbl genes were in the UniBind ChIP-seq database. We also found predicted or ChIP-seq-detected TFBS for TFs that are highly specific for either myoblasts (*MYOD*, *MYF5*, *MYF6*, and *MYOG*) or cerebellum (*ETV1*, *NEUROD1*, *NEUROG1*, *OTX2*, or *PAX6*; Figure S11).

Lastly, for the Myob/Cbl genes that encode TFs, we also examined relative transcription profiles in human fetuses and embryos using the EmAtlas [29] (which does not have brain data other than spinal cord). Four of these genes are expressed preferentially in fetal SkM and spinal cord (*ZIC1*, *PAX3*, *VAX2*, and *LBX1*). *EN2*, like *ZNF556* (Figure 4), is preferentially expressed in fetal SkM, oocytes, and pre-implantation embryos (Figure S12).

## 3. Discussion

From a genome-wide examination of transcriptomics and epigenomics, we identified twenty genes (Myob/Cbl genes) that have a strong preference for transcription in myoblasts (mesodermally derived SkM progenitor cells) and cerebellum, a highly dissimilar cell population (ectodermally derived). The co-expression of genes in myoblasts and cerebellum, rather than in other brain regions (Table S3), reflects the much higher number of genes preferentially expressed in cerebellum than elsewhere in the brain ([16,44] and Table S2). In contrast, myoblasts were not unusual compared to several other progenitor cell strains in having a small subset of genes preferentially expressed in both that cell culture (HUVEC, NHEK, or NHLF) and cerebellum (Table S5). However, only Myob/Cbl genes had a significant ontological association with TFs (Table S5, *ZIC1*, *EN2*, *PAX3*, *VAX2*, *LBX1*, and *ZNF556*), which suggests a special transcriptional relationship for myoblasts and cerebellum for a small number of genes. Among the twenty Myob/Cbl genes, the strongest association of myoblast DNA hypomethylation with gene expression was seen for *ZNF556.* Very little is known about its function, but the encoded protein has the structural hallmarks of KRAB zinc finger TFs, which often act as repressor proteins [34,45]. The *ZNF556* promoter overlaid a Myob-hypom DMR, which was highly methylated in all 17 examined non-expressing ENCODE cell populations and hypomethylated in promoter chromatin specifically in all *ZNF556*-expressing cell populations (Figure 2 and Figure 3). Its hypomethylation and expression in HepG2, a hepatocarcinoma cell line, provides an example of cancer DNA hypomethylation coupled with expression of a gene normally active in a small number of very different cell types.

In predicting the relationship of promoter DNA methylation to gene expression, the CpG content of the promoter region is critical [46]. The *ZNF556* Myob-hypom DMR (TSS −0.7 to +1.3 kb) almost fits the UCSC Genome Browser’s definition of a CGI except that its observed CpG density/expected CpG density was 0.58 instead of >0.6. This promoter DMR would be classified as an intermediate CpG promoter by the definition of Weber et al. [46]. CGI promoters are predominantly constitutively unmethylated or very lowly methylated. Normal or disease-acquired methylation of CGI promoters and intermediate-to-high CpG promoters is strongly associated with transcription repression in human cells [46,47,48]. Therefore, it is likely that methylation of the *ZNF556* DMR in vivo helps silence it. To test this, we determined the effect of DNA methylation on *ZNF556* promoter activity in reporter gene assays. In vitro methylation of cloned *ZNF556* DMR sequences reduced promoter activity by about half (Figure 3). Downregulation in vivo by DNA methylation might be much stronger because we tested the effect of DNA methylation on constructs that did not include the further downstream, CpG-enriched parts of the DMR (Figure 3). We conclude that the *ZNF556* DNA hypomethylation in vivo coupled with promoter chromatin at the promoter region enables tissue/cell-specific transcription of this gene.

Unexpectedly, the region immediately upstream of the *ZNF556* TSS (TSS −0.6 to +0.1 kb) did not suffice for appreciable promoter activity in the reporter gene assays even though it overlapped the only prominent DNase-seq peak at the promoter region in *ZNF556*-expressing samples. Our transfection data indicates that for appreciable promoter activity, this expression-associated open-chromatin region has to cooperate with adjacent upstream or downstream sequences. In myoblasts and cerebellum, the adjacent sequences are either hypomethylated or they are CpG-methylated in a region of low CpG density (Figure 3). The unusually high concentration of DNA repeats in the TSS-upstream region, especially the LTRs (Long Terminal Repeats, Figure 3A, bottom), might influence the activity and/or the methylation status of the promoter [49].

The hypomethylation of the *ZNF556* promoter region in germline cells can help explain the finding that high methylation of its promoter is the default state in non-expressing cells. The *ZNF556* promoter region was hypomethylated in ovarian stromal cells, oocytes, spermatogonia, early spermatids as well as zygotes, two-cell embryos, and four-cell embryos (but not morula), and all of these cell populations contain *ZNF556* RNA (Figure 4; [28,29]). The high level of methylation of the *ZNF556* promoter in most somatic cell types is unlike the strong correlation of CpG-rich promoters having little or no DNA methylation in normal somatic cell types [50]. The best characterized group of genes that are an exception to this general finding is the set of genes specifically expressed in the germline [46]. Some CpG-rich promoters are highly methylated in somatic tissues and hypomethylated in sperm [51] as a result of large losses in DNA methylation from many parts of the genome in mammalian primordial germ cells, which ultimately give rise to both sperm and oocytes [52]. For some of the above-mentioned cell types, e.g., zygotes, *ZNF556* promoter hypomethylation might be a memory of former transcriptional activity and possibly a poised state for future activation. This relationship of *ZNF556* expression to gametogenesis and pre-implantation embryos suggests a role for *ZNF556* in pre-implantation embryos that might be shared by its neighbor *ZNF555*, as evidenced by the even higher enrichment of *ZNF555* RNA in zygote, two-cell, and four-cell embryos than was seen for *ZNF556* (Figure 4). Therefore, we propose that during evolution, first, *ZNF556* was expressed just in the germline and possibly the preimplantation embryo and only later was there repurposing of the associated epigenetics in myoblasts and cerebellum to extend the tissue/cell specificity of this TF and its functionality.

Two other Myob/Cbl genes, *CDH15* and *TRIM72*, had DNA hypomethylation in the promoter region that is likely to help contribute to the high transcription of these genes in myoblasts and cerebellum (Table 1). Previously, we demonstrated that a *CDH15* intragenic CGI displaying RRBS-detected myoblast hypermethylation is a methylation-inhibited cryptic promoter [13]. However, because of the limited coverage by RRBS, that study did not find the long region of low DNA methylation that stretches from upstream of the *CDH15* TSS to far downstream into intron 1 specifically in myoblasts and cerebellum, as seen in WGBS profiles (Figure 1). The myoblast- and cerebellum-hypomethylated DNA regions overlapping the *CDH15* and *TRIM72* promoters are transcription-associated extensions of adjacent, constitutively unmethylated regions that overlap CGIs (Figure 1 and Figure 5).

*TRIM72* presents the unusual example of a gene with a Myob-hypom DMR at its promoter and a Myob-hyperm DMR overlapping the promoter of its intronic, protein-coding gene, *PYDC1* (Figure 5)*. PYDC1* resides in intron 2 of *TRIM72* and is positioned antisense to *TRIM72*, its host gene. Unlike *TRIM72*, *PYDC1* was silenced in myoblasts and SkM but selectively expressed in cerebellum (TPM ratio 4.7 for cerebellum vs. the average of 10 other brain regions). RNA levels for *TRIM72* are almost 4-fold lower in cerebellum than in SkM, which might be due, in part, to dampening of *TRIM72* transcription in cerebellum due to the clashing movement of RNA polymerase complexes from the *PYDC1* TSS and the *TRIM72* TSS. This dichotomous expression of two overlapping genes in cerebellum and myoblasts is probably achieved, in part, by the Myob/SkM-hyperm DMR that extended from the *PYDC1* promoter region. In contrast, cerebellum lacked DNA methylation at and around *PYDC1*, as did most tissues with a silent *PYDC1* gene. This indicates that the *PYDC1* CGI promoter is constitutively unmethylated regardless of expression status, with the exceptions of myoblasts and SkM (Figure 5). This SkM-lineage hypermethylation overlaps enhancer chromatin or mixed enhancer/promoter chromatin. The hypermethylation probably helps establish or maintain an intragenic *TRIM72* enhancer in the SkM lineage at a region that is an active, unmethylated *PYDC1* promoter in cerebellum and repressed chromatin in most other cell populations.

Among the Myob/Cbl genes, Myob-hyperm DMRs were more frequent than Myob-hypom DMRs (Table 1). Some of this differential methylation may help direct alternative promoter usage for *ANK1*, *CNPY1*, *DOK7*, and *MCF2L* (Figure 6, Figures S7, S9, and S10). The alternate promoter usage for these genes not only changes the polypeptide that the gene encodes, but also, in the case of *ANK1*, might affect the efficiency of production of a co-transcribed intronic miRNA miR-486 (Figure 6). This miRNA plays pivotal roles in myogenesis and is important for normal heart formation in mice [37]. Moreover, it is being considered for therapeutic disease modulation because its upregulation in dystrophic mouse models can reduce symptoms of muscular dystrophy [37]. In addition, in some cancers, miR-486 is an oncogenic marker and may play a role in oncogenesis [53,54]. DNA hypermethylation at multiple *ANK1* CGI promoter regions is associated with cancer-linked changes in miRNA levels [53]. However, the epigenetics of the myoblast/SkM/heart promoter in cancers has received little attention probably because it does not overlap aCGI. There might be cancer-associated hypomethylation of this *ANK1* promoter, which could influence miR-486 levels as well as *ANK1* isoform abundance ratios.

Some Myob/Cbl genes exhibit only moderately low steady-state levels of RNA in myoblasts but much higher levels in cerebellum (Table 1). Five of these genes (*KCNJ12*, *ST8SIA5*, *ZIC1*, *EN2*, and *VAX2*) displayed Myob-hyperm DMRs upstream and/or downstream of their core promoter region that were missing in cerebellar neurons (Table 1, Figures S2, S4–S6). The DMRs probably downmodulate gene expression in myoblasts vs. in cerebellum. In a previous study of *TBX15*, a TF-encoding gene preferentially expressed in myoblasts and SkM but not in any brain region, we used reporter gene assays and in vitro methylation to demonstrate that enhancer and promoter activity of Myob-hyperm DMR sequences upstream or downstream of its TSS was strongly suppressed by DNA methylation [17]. In the case of *PAX3*, myoblast and cerebellum hypermethylation upstream and downstream of its promoter in both myoblasts and cerebellum is probably helping to keep transcription low in both cell populations (Table 1, Figure 7).

In addition to regulating usage of alternative promoters, directing usage of intronic promoters, and silencing cryptic promoters, intragenic hypermethylation in transcribed genes can facilitate movement of the RNA polymerase complex across the gene body of actively transcribed genes to regulate alternate splicing [55,56]. It has also been proposed that such intragenic methylation associated with transcription may be simply a consequence of the recruitment of DNMT3 enzymes’ PWWP domain by H3K36me3. Twelve of the Myob/Cbl genes had intragenic Myob-hyperm DMRs but only three of them (*CDH15*, *ANK1*, and *MCF2L*) had DMRs that overlapped H3K36me3-enriched chromatin in myoblasts (Txn-chrom, Figure 1, Figure 6 and Figure S9). Another mechanism for intragenic or intergenic DNA hypermethylation being positively associated with gene expression is that DNA methylation may decrease the spread of repressive H3K27me3-enriched chromatin in many chromatin/DNA contexts [57,58]. Although epigenetic profiles suggested that this is not the function of most of the examined DNA hypermethylation at Myob/Cbl genes, it might be the case for the *VAX2* TSS-downstream DNA hypermethylation in myoblasts and cerebellum (Figure S5).

A caveat in our study is that DNA methylation levels at *cis*-regulatory elements can vary with the exact nature of the cells or tissues studied (physiology, cell composition, age, and health status of the donor, and for SkM, the muscle location and fiber type [8,59,60,61]). However, these changes are usually less than the strong differences in DNA methylation that are tissue specific. Another caveat is that WGBS does not distinguish between genomic 5-methylcytosine (5mC) and 5-hydroxymethylcytosine (5hmC), which often have different biological effects [62,63]. This is not a complication for Myob-hyperm DMRs because we found that myoblast cell strains have little or no 5hmC at tested CpG sites in 13 examined myoblast RRBS-delineated DMRs, including one in the *CDH15* intragenic Myob-hyperm DMR overlapping a cryptic intragenic promoter [13]. This is consistent with the loss of genomic 5hmC reported upon passage of mouse embryonic fibroblast cell strains [64]. In the case of tissues, especially cerebellum, how much of the WGBS signal for DNA methylation is actually 5hmC is an important consideration [13,63,65]. For example, we previously found that SkM and cerebellum at a tested CpG in the *CDH15* Myob/SkM-hyperm DMR has about twice as much 5hmC as 5mC [13].

An illustration of the need to consider the 5hmC content of hypermethylated regions in cerebellum comes from the important findings of James et al. about the homeobox TF EN2 [59,66]. EN2, like several other Myob/Cbl genes (Table S4), has been associated with neurological syndromes. The studies of James et al. suggest a role for EN2 in the autism spectrum disorder in addition to its pivotal contributions to cerebellum development [67]. They found that a 0.15 kb region ~3 kb upstream of the *EN2* TSS had more 5hmC as well as 5mC in patient samples than in matched controls. These epigenetic changes were positively associated with more *EN2* RNA and protein. We observed that this region is partially methylated in normal cerebellum in contrast to little or no methylation in other studied tissues and cell types (Figure S7E,G). Moreover, Szulwach et al. [68] found that the ~4–8 kb region upstream of the mouse *En2* gene in cerebellum contains peaks of 5hmC overlapping a previously identified embryonic enhancer for the *En2* gene. Several of the human cerebellum-hypermethylated regions that we observed upstream of the *EN2* promoter are adjacent to DNase-seq peaks. These regions might help demarcate an enhancer or, if they contain a sufficient percentage of 5hmC, contribute to enhancer activity, or just counteract binding of the repressive MECP2 protein [59,67,69].

For some of the Myob/Cbl genes, there are apparent functional relationships between myoblasts and cerebellum. Nine of these genes (*ANK1*, *CDH15*, *DOK7*, *FNDC5*, *MCF2L*, *TRIM72*, *CHRD*, *KCNJ12*, and *PTPRR*) encode proteins localized mostly or in part to the plasma membrane, and the first six of these were expressed at moderate to high levels in both myoblasts and cerebellum (Table 1 and Table S5). Regulation of cell–cell interactions is critical for controlling neuronal function [70] as well as for regulating myoblast fusion [71,72]. An example of a Myob/Cbl gene with known myoblast and brain functions for one of these plasma membrane-associated Myob/Cbl gene-encoded proteins is the above-mentioned *CDH15*. Like other classical transmembrane cadherins, CDH15 is implicated in signal transduction downstream of its mediation of homotypic cell–cell contacts [72]. CDH15 is one of the cadherins that can be involved in fusion of myoblasts to form multinucleated myotubes via its role in cell–cell interactions [23,71,72]. It is also implicated in intellectual functions from studies of mutationally linked intellectual disability syndromes in which the mutations alter cell–cell contacts [73,74].

Like 11 other Myob/Cbl genes, CDH15 is much more highly expressed in myoblasts than in SkM (Table S4). The yet higher expression of *CDH15* in myoblasts than in SkM may be due to an extensive role of CDH15 in myoblast fusion at the cell membrane compared to a more limited role of the cadherin in SkM, especially at the satellite cell-myofiber contact point [71,72]. Very many differences in the transcriptomics and epigenomics of myoblasts (mononuclear SkM progenitor cells) and myocytes (extensively multinucleated cells in SkM fibers with unique SkM cellular and organ characteristics) are expected and have been characterized (e.g., [42]). There was not only diversity in ratios of Myob to SkM expression but also in functions of Myob/Cbl genes (Table S4). Nonetheless, a few common features were seen among gene subgroups, e.g., roles in cell shape changes or cell mobility. The preferential expression of many of the Myob/Cbl genes in myoblasts and cerebellum probably reflects co-option of varied functions from embryonic to postnatal stages and from the mesodermal to ectodermal cell lineages or vice versa.

One functional relationship shared by multiple Myob/Cbl genes is that six of them (*ZNF556*, *EN2*, *ZIC1*, *PAX3*, *LBX1*, and *VAX2*) encode TFs, four of which contain homeoboxes. Both “transcription factor activity” and “homeobox” categories were significantly overrepresented among the twenty Myob/Cbl genes (Table S5). Highly regulated modulation of levels of expression at different times in development or in response to physiological changes is especially important for genes encoding such proteins [67,75] and often requires changes in epigenetics. Some of the precise transcriptional regulation of Myob/Cbl genes by promoters and enhancers is likely to involve binding of TFs that are specific for either the SkM or the neural lineages (Figure S11). In contrast, TFs encoded by Myob/Cbl genes have dual myoblast and cerebellum specificities. Two Myob/Cbl genes, *PAX3* and *LBX1*, code for TFs that are involved in both skeletal muscle development and neuronal differentiation (Table S4; [72,76,77,78]). *PAX3* is implicated in the regulation of transcription of three Myob/Cbl genes, *EN2*, *ANK1*, and *LBX1* [79,80]. LBX1 or EN2 TFs were predicted to bind to Myob-hypom DMRs at *ZNF556* and *ANK1* promoters (Figure S11). We propose that epigenetic control of expression of TF-encoding Myob/Cbl genes in these two dissimilar cell populations not only helps regulate their expression, but also, in *trans*, regulates the tissue/cell-specificity of other Myob/Cbl genes.

## 4. Conclusions

We identified twenty human genes (Myob/Cbl genes) preferentially expressed in myoblasts and cerebellum, two highly divergent cell populations. Similarities in cell/tissue-specific promoter hypomethylation between myoblasts and cerebellum vs. other cell cultures or tissue types correlate with cell/tissue expression specificity for several of the genes. In addition, differences in DNA methylation between myoblasts and cerebellum may contribute to modulating relative expression levels or directing alternative promoter usage for some Myob/Cbl genes. The six Myob/Cbl genes that encode transcription factors may help drive the specific transcription profiles of the other genes preferentially expressed in myoblasts and cerebellum. Our study shows how epigenetic analyses in many different cell populations for genes that share highly specific and unexpected cell/tissue specificity can help in understanding normal differentiation and disease-linked changes in gene expression.

## 5. Materials and Methods

### 5.1. Transcriptomics

The set of human genes considered for identifying Myob/Cbl genes came from the intersection of the GTEx RNA-seq database set for tissues and an ENCODE dataset for cell cultures [16,42,43]. Genes without at least one tissue and at least one cell culture having a TPM or FPKM of ≥1 as well as mitochondrially located genes and most non-coding genes were removed to give 13,847 genes. These were first sorted for myoblast specificity using non-strand-specific cell culture RNA-seq data from ENCODE (ENCODE Regulation Transcription Track Settings (ucsc.edu)) for the six available cell cultures that were not derived from cancers. These are myoblasts, NHLF, LCL (GM12878), HUVEC, NHEK, and ESC (H1). Of these cultures, only the LCL is a transformed cell line (EBV-transformed B cells). Myoblast-preferential expression was defined as genes with a myoblast FPKM divided by the average of the FPKM for the other cell-types of ≥5 and an FPKM for myoblasts ≥ 1. Cerebellum-preferential expression was assessed with RNA-seq data for human tissues in the GTEx database (GTEx Portal). The criteria were an expression ratio of ≥5 for cerebellum TPM (a median value from 241 biological replicates) vs. the median TPM of 10 tissues from other brain regions, a ratio of ≥5 for cerebellum vs. the average of 41 non brain tissues, and a TPM of ≥1 for cerebellum (Table S2). For the 12 genes where the average TPM of non-brain tissues was 0, two of those genes had their maximum TPM in cerebellum and were added to the set of cerebellum-preferentially transcribed genes. Another bulk RNA-seq database used to characterize selected genes was the strand-specific ENCODE RNA-seq for cell cultures (ENC RNA-seq CSHL Long RNA-seq Track Settings (ucsc.edu)). CAGE profiles of cell cultures available at the UCSC Genome Browser RIKEN CAGE Loc Track Settings (ucsc.edu) were examined where indicated. For comparison of myoblast and myotube expression our RNA-seq data were used [27]. In addition, three single-cell RNA-seq (scRNA-seq) databases used for analysis of Myob/Cbl genes were HPA (The Human Protein Atlas, [28]) for postnatal cells, Fetal Gene Atlas Tracks (ucsc.edu) for fetal cells [26], and EmAtlas, a compilation of scRNA-seq data from human early embryonic cells, oocytes, and mid-gestation fetal tissues (EmAtlas (imu.edu.cn)) [29].

### 5.2. Epigenomics

The chromatin epigenomics data (chromatin state segmentation and H3K27ac) were from the Roadmap Epigenomics Project [19], as previously described [17], and visualized in the UCSC Genome Browser (https://www.genome.ucsc.edu/, accessed on 28 August 2023). Coordinates are in hg19, unless otherwise stated; tracks only in hg38 coordinates were lifted over to hg19. DNase-seq data was from ENCODE (ENC DNase/FAIRE Duke DNaseI HS Track Settings (ucsc.edu)). TF binding analyses used UCSC Genome Browser track hubs for JASPAR TFBS prediction (JASPAR CORE 2022, minimum score, 400) and the UniBind database (for TFBS of interest, including CTCF) based upon ChIP-seq results and the TFBS site location within the predicted region (UniBind 2021 Permissive). C2C12 MyoD sites are sequences homologous to C2C12 mouse myoblast sequences shown to bind MyoD upon ChIP-seq [81]. WGBS data were obtained from different sources available on the UCSC Genome Browser, with low methylated regions displayed, where available (http://smithlabresearch.org/software/methbase/, assessed on 28 August 2023–8 December 2023). We generated myoblast WGBS and EM-seq profiles from well-characterized primary myoblast cultures derived from gastrocnemius muscle as previously described [17,42] and included them as well as a publicly available cerebellum WGBS profile [18] (GSM5652231_Cerebellum-Neuron-Z000000TB.hg38.bigwig) as custom tracks at the UCSC Genome Browser. SkM and heart refer to psoas muscle and left ventricle, respectively, unless otherwise specified. Myoblast DMRs were determined by comparing the EM-seq profiles from three biological replicates to WGBS profiles of foreskin fibroblasts, HMEC, IMR90 (fetal lung fibroblast cell line), ESC, and adipose-derived mesenchymal stem cells induced to differentiate to adipocytes and prostate epithelial cells as previously described [17]. Also as previously detailed [17,82], SkM DMRs were determined from psoas SkM WGBS vs. WGBS from heart (left ventricle), aorta, monocytes, lung, and subcutaneous adipose tissue. This tissue set includes the three main types of muscle tissue (striated skeletal, striated cardiac, and smooth muscle) and two diverse tissue types. The threshold for DMR attribution was absolute methylation differences of ≥0.35. Myoblast methylome profiles from WGBS and EM-seq from myoblast samples were very similar, as illustrated in Figure S1C.

### 5.3. Reporter Gene Assays

Reporter gene constructs were prepared by overlap extension PCR (Table S6) or by using the Gibson assembly kit (NEBuilder HiFi Assembly, New England Biolabs, Ipswich, MA) and a CpG-free plasmid vector (pCpGfree-Lucia, InvivoGen) as previously described [17]. The recombinant plasmid structure was checked by partial DNA sequencing and restriction site analysis. Transfection into C2C12 or MCF-7 cells utilized a lipid-based reagent (Fast-forward protocol, Effectene reagent, Qiagen, Germantown, Md). As a reference for transfection efficiency, pCMV-CLuc 2 (New England Biolabs) encoding the Cypridina luciferase was co-transfected with the test construct. About 48 h after the transfection, Lucia and Cypridina luciferase activity was quantified by bioluminescence from aliquots of the cell supernatant (BioLux Cypridina Luciferase assay kit, New England Biolabs; Quanti-Luc, InvivoGen, San Diego, CA). Reference plasmid-normalized luciferase activity was from the average of three independent transfections. Methylation of the plasmids was targeted just to the *ZNF556* inserts, which were the only CpG-containing sequences, by incubating the DNA construct (1 μg) with 4 units of SssI methylase and 160 μM S-adenosylmethionine (New England Biolabs) for 4 h at 37 °C or mock methylating by similar incubation but in the absence of S-adenosylmethionine. A plasmid construct that contained three BstUI CGCG sites was similarly methylated and shown thereafter to be fully resistant to BstUI cleavage.

## Figures and Tables

**Table 1:** Genes preferentially expressed in myoblasts and cerebellum.

	FPKM or TPM (Expression Ratio ≥5) ^a^		No. of Myob DMRs ^b^		Probable Function of Differential Methylation ^c^	
Gene	Myob	Cbl	Hypom	Hyperm	Myob DMRs	Cbl Hypom or Hyperm
*ZNF556*	12 (1076)	2 (27)	1	0	Prom hypom allowing txn ^d^	Prom hypom allowing txn ^d^
*CDH15*	164 (560)	141 (1441)	1	2	Prom dnstm hypom ↑ txn ^d^	Prom dnstm hypom ↑ txn ^d^
*TRIM72*	43 (127)	12 (53)	1	1	Prom hypom ↑ txn ^d^; hyperm repr intronic *PYDC1*	Prom hypom ↑ *TRIM72* txn ^d^ and intronic *PYDC1*
*ANK1*	29 (15)	85 (25)	2	1	Alt prom usage	Different prom use from Myob
*MCF2L*	21 (5)	110 (9)	2	3	Alt prom usage ^d^; many RNA splicing isoforms	Alt prom and splicing ^d^; hypom ↑ txn from enhs
*DOK7*	45 (220)	21 (27)	0	1	Alt prom usage ^d^	Alt prom usage ^d^
*CNPY1*	2 (39)	26 (158)	0	2	Alt prom usage	Alt prom usage
*KCNJ12*	5 (6)	81 (40)	0	3	Prom-dnstm hyperm ↓ txn	Prom-adjacent hypom ↑ txn
*ST8SIA5*	4 (6)	51 (14)	0	1	Prom-upstm hyperm ↓ txn	Prom-dnstm hypom ↑ txn
*ZIC1*	5 (39)	311 (57)	0	14	Prom-upstm/dnstm hyperm ↓ txn and repressing adj *ZIC4*	Whole-gene hypom ↑ txn of both *ZIC1* and *ZIC4*
*VAX2*	4 (6)	19 (15)	0	7	Prom-upstm hyperm ↓ txn; Intron-1 hyperm may block formation of repr chrom ^d^	Intron-1 hyperm may block formation of repr chrom ^d^
*EN2*	3 (56)	68 (201)	0	4	Prom-upstm/dnstm hyperm may ↓ txn	Hyperm far upstm and dnstm of 7 kb *EN2* may ↑ txn
*LBX1*	1 (230)	3 (78)	0	4	Hyperm upstm/dnstm of 2 kb gene may ↑ txn	Methylation profile similar to those of most tissues
*PAX3*	1 (23)	4 (28)	0	11	Prom-upstm/dnstm hyperm ↓ txn ^d^	Prom-upstm/dnstm hyperm ↓ txn ^d^
*CHRD*	4 (7)	243 (22)	1	0	Intergenic hypom may precede Enh formation	Hypom at different intergenic regions ↑ txn
*FNDC5*	64 (88)	109 (8)	0	0	NA	3′ gene hypom ↑ txn
*PLCB4*	54 (22)	57 (18)	0	0	NA	Prom-dnstm hypom ↑ txn
*MPP4*	9 (9)	3 (16)	0	0	NA	Uncertain
*PTPRR*	2 (6)	22 (9)	0	0	NA	Alt prom usage
*IL11*	6 (6)	6 (14)	0	0	NA	Uncertain

^a^ Expression ratio, FPKM for myoblasts vs. average FPKM for other cell cultures or TPM for cerebellum vs. average for ten other non-cerebellum brain regions (Tables S1 and S2). Myob, myoblasts; Cbl, cerebellum; hypom, hypomethylation; hyperm, hypermethylation. ^b^ Number of DMRs; the promoter hypomethylation in myoblasts for *CDH15* was seen as a long specific LMR (Figure 1C); the cryptic promoter activity of the intragenic *CDH15* hyperm DMR was previously reported [17]. ^c^ Prom, promoter; txn, transcription; ↑, upregulates; ↓, downmodulates; alt, alternative; Enh, enhancer chromatin; dnstm, downstream; upstm, upstream; adj, adjacent; repr chrom, repressive chromatin. ^d^ Both myoblast and cerebellum share hypomethylation or hypermethylation and similar associations with gene expression.

## Data Availability

Data can be found in Supplementary tables and the referenced websites.

## Supplementary Materials

The following supporting information can be downloaded at: https://www.mdpi.com/article/10.3390/epigenomes8010004/s1, Figure S1. The *ZNF556* neighborhood contains other KRAB-ZNF genes that, unlike *ZNF556*, are not expressed preferentially in myoblasts or cerebellum. Figure S2. Promoter hypomethylation in cerebellum is associated with upregulated expression of *ZIC1* while, in myoblasts, hypermethylation of the neighboring gene, *ZIC4*, is associated with repression of its expression. Figure S3. Intragenic hypomethylation in *CHRD* is associated with its upregulation in cerebellum. Figure S4. Cerebellar hypomethylation and myoblast hypermethylation in *KCNJ12* are associated with up- or down regulation of this gene. Figure S5. Hypermethylation upstream of the *VAX2* promoter in myoblasts and in intron 1 in myoblasts and cerebellum is associated with *VAX2* preferential expression both cell populations. Figure S6. The promoter region of *ST8SIA5* in cerebellum is more extensively hypomethylated than that of brain prefrontal cortex. Figure S7. *EN2* and *CNPY1* have differential DNA methylation associated with tissue-specific expression levels and display cerebellar DNA hypermethylation in a region previously associated with autism-linked hypermethylation. Figure S8. Extensive upstream hypermethylation in myoblasts and SkM and hypomethylation in cerebellum is associated with enhanced *LBX1* expression in muscle. Figure S9. Myoblast DMRs in *MCF2L* likely affect promoter usage in cerebellum and myoblasts. Figure S10. An intragenic region of myoblast and cerebellum hypermethylation is associated with repression of alternative promoter usage for *DOK7* in these cell populations. Figure S11. Transcription factor (TF) binding at four Myob/Cbl gene regions showing hypomethylation in both myoblasts and cerebellum. Figure S12. Relative mRNA levels for five of the Myob/Cbl genes in embryonic tissues or pre-implantation embryos. Table S1. Genes preferentially expressed in myoblasts. Table S2. Genes with preferential expression in cerebellum. Table S3. Genes preferentially expressed in various brain regions. Table S4. Gene Ontology (GO) associations for individual genes preferentially expressed in both myoblasts and cerebellum and myoblast–cerebellum biological relationships. Table S5. Gene ontology analyses on the sets of genes with cerebellum preferential expression as well as preferential expression in one of each of the six cell cultures. Table S6. Oligonucleotides used for fusion cloning of 5′ *ZNF556* sequences into CpG-free Luciferase reporter vectors. References [83,84,85,86,87,88,89,90,91,92,93,94,95,96,97,98,99,100] are cited in the Supplementary Materials.
