# Supplementary material for: Epigenetics of Genes Preferentially Expressed in Dissimilar Cell Populations: Myoblasts and Cerebellum
Source: Epigenomes. 2024 Jan 26;8(1):4. doi: 10.3390/epigenomes8010004 (PMC10885033; doi:10.3390/epigenomes8010004)
Supplement: Supplementary file 1 [file epigenomes-08-00004-s001.zip › Ehrlich_Cerebellum_Myob_Pref_Expr_Genes_Suppl Figs_EN_F_1_22_23.pdf]

Supplementary Figures S1 to S12 for

Epigenetics of genes preferentially expressed in dissimilar cell populations: Myoblasts and cerebellum

Melanie Ehrlich, Kenneth C. Ehrlich, Michelle Lacey, Carl Baribault, Sagnik Sen, Pierre Olivier Esteve, Sriharsa Pradhan

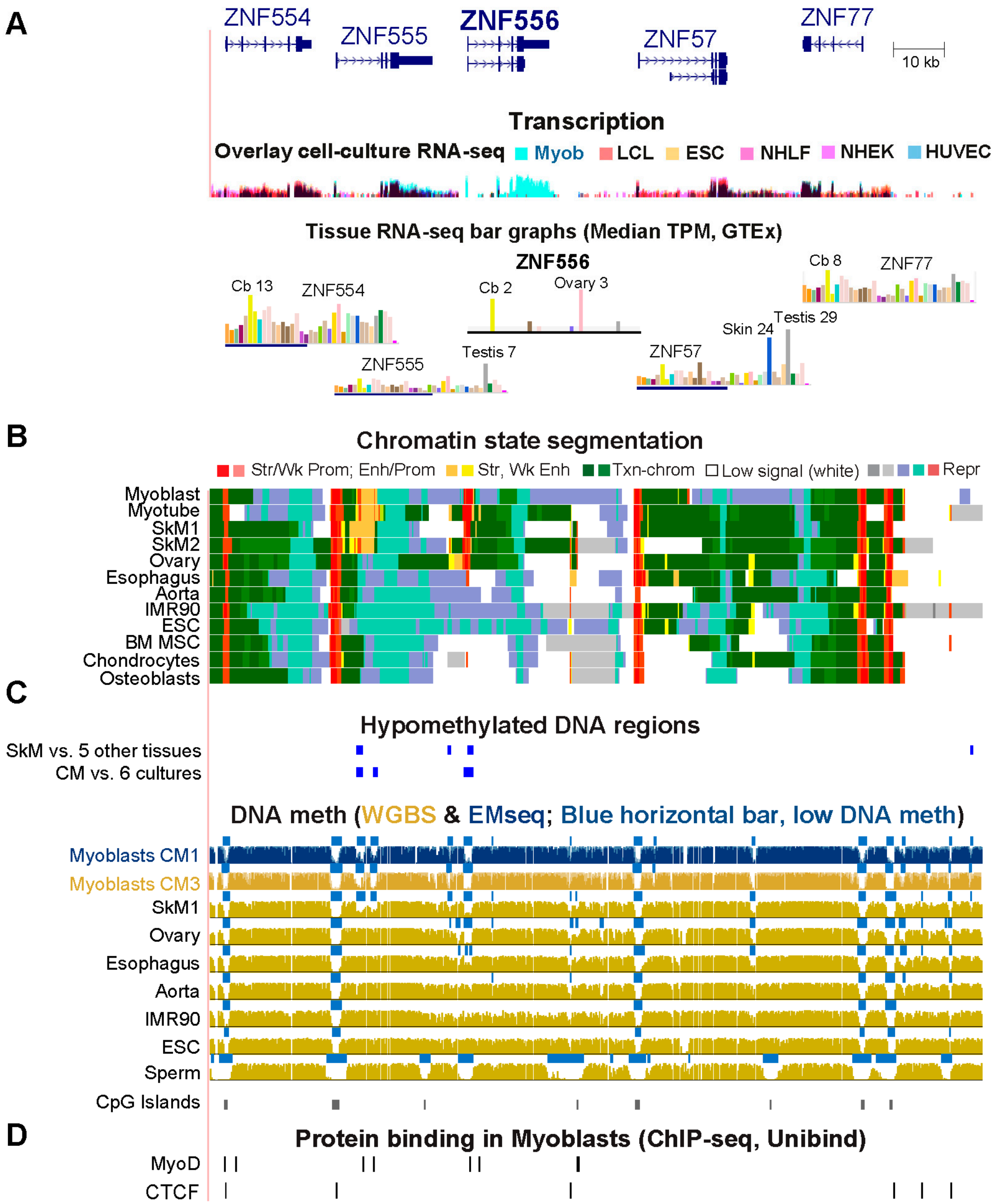

Figure S1. The *ZNF556* neighborhood contains other KRAB-ZNF genes that, unlike *ZNF556*, are not expressed preferentially in myoblasts or cerebellum. *ZNF554* is implicated in modulating trophoblast cell invasion [1] and *ZNF57* (a paralog of *ZNF555*) in normal hearing [2] while the roles of *ZNF556* and *ZNF555* in normal development are uncertain. (**A**) The region shown is chr19:2,816,866-2,968,497. RNA-seq for cell cultures is shown as log transform of the overlay signal and for 37 tissues as bar graphs with relative heights (linear scale) from GTEx median values. (**B**) Chromatin state segmentation (18-state) is based on key histone methylation and acetylation profiles (Roadmap Epigenomics Project). (**C**) Significant myoblast DMRs and SkM (psoas) DMRs and WGBS and EM-seq for myoblasts; two different myoblast cell cultures, CM3 and CM1, were used for the WGBS and EM-seq, respectively). Blue horizontal bars are regions of significantly lower methylation than in the whole genome for the same sample. All tracks are horizontally aligned in this and other figures (pink line) and are from the UCSC Genome Browser (http://www.genome.ucsc.edu/) hg19 reference genome. (**D**) CTCF and MyoD binding sites are from UniBind. Abbreviations: Cb, cerebellum (in GTEx bar graph); Str, strong; Wk, weak; Prom, promoter; Enh, enhancer; Txn-chrom, chromatin with the H3K36me3 indicative of active transcription; Low signal, negligible signal for H3K27ac or me3, H3K4me1 or 3, H3K9me3, or H3K36me3; Repr, repressed; Myob, myoblasts; LCL, lymphoblastoid cell line (GM12848); ESC, human embryonic stem cells (H1); NHLF, lung fibroblasts; NHEK, normal human erythroid keratinocytes; HUVEC, human umbilical cord epithelial cells; SkM1, skeletal muscle (psoas); SkM2, female leg SkM; IMR90, fetal lung fibroblasts; BM-MSC, bone marrow-derived cultured mesenchymal stem/stromal cells.

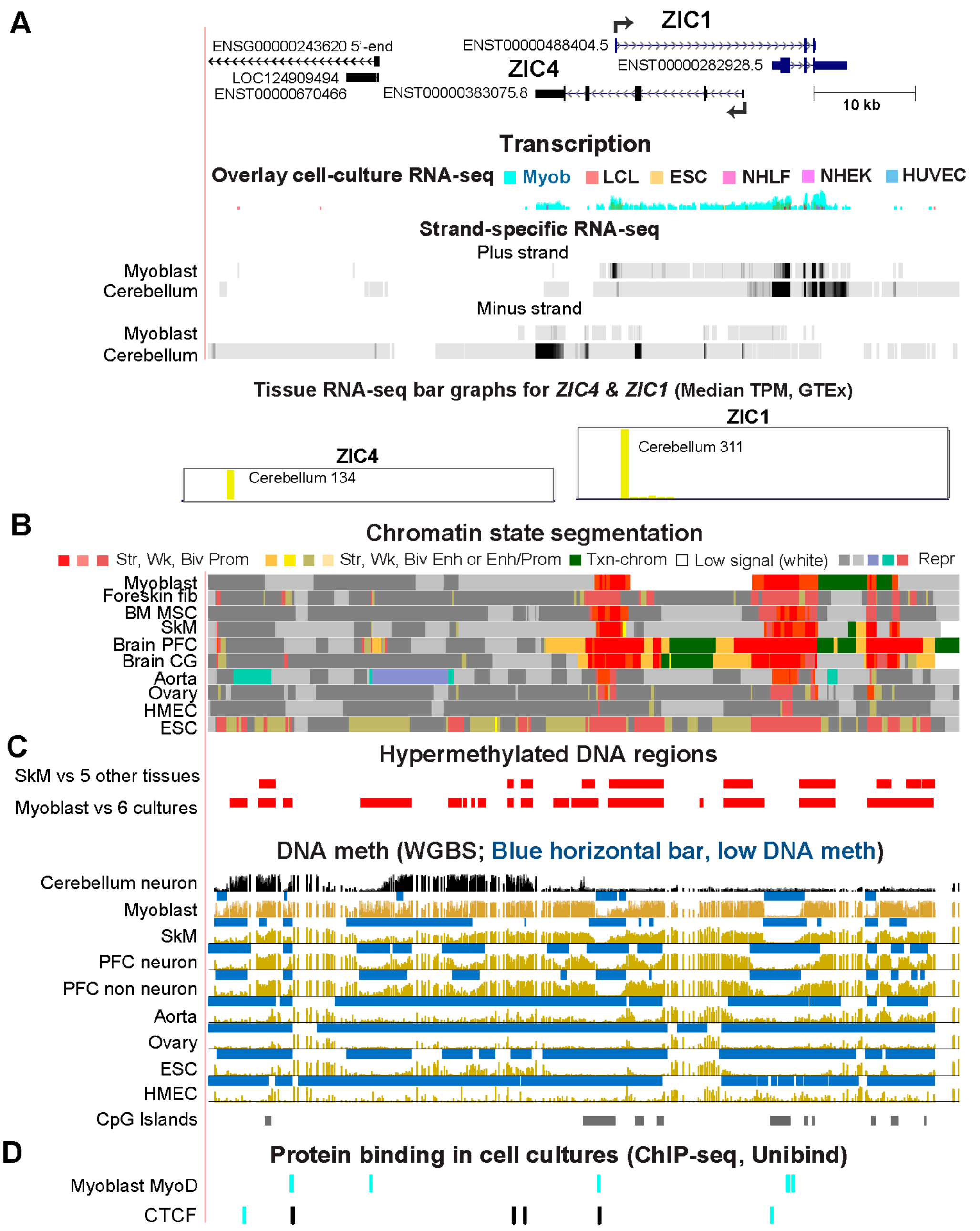

Figure S2. Promoter hypomethylation in cerebellum is associated with upregulated expression of *ZIC1* while, in myoblasts, hypermethylation of the neighboring gene, *ZIC4*, is associated with repression of its expression. *ZIC1* encodes a transcription factor that is involved in neurogenesis, mainly in the development of the cerebellum and spinal cord [3,4]. Its altered expression is associated with several brain diseases [5,6]. *ZIC1* is also implicated in regulation of myogenesis [7]. The single RefSeq Curated isoform for *ZIC1* and the RefSeq Select isoform of *ZIC4* are shown at chr3:147,071,570-147,145,591. Panels **A** to **D** are similar to those of Figure S1 except that Panels **C** and **D** are combined. Panel **A** includes strand-specific RNA-seq for cerebellum from ENCODE/Washington University (setting 0-0.5) and Myob (Long RNA-seq from ENCODE/Cold Spring Harbor Laboratory, vertical viewing range, 0-150 for the plus strand and 0-30 for the minus strand). Abbreviations: Fib, fibroblasts; Brain CG, cingulate gyrus; PFC, prefrontal cortex; HMEC, mammary epithelial cell culture. Panel **D**, blue, the CTCF site was seen in myoblasts but not in HMEC or ESC; black, the CTCF binding sites that were seen in myoblasts and ESC or HMEC. SkM, psoas skeletal muscle.

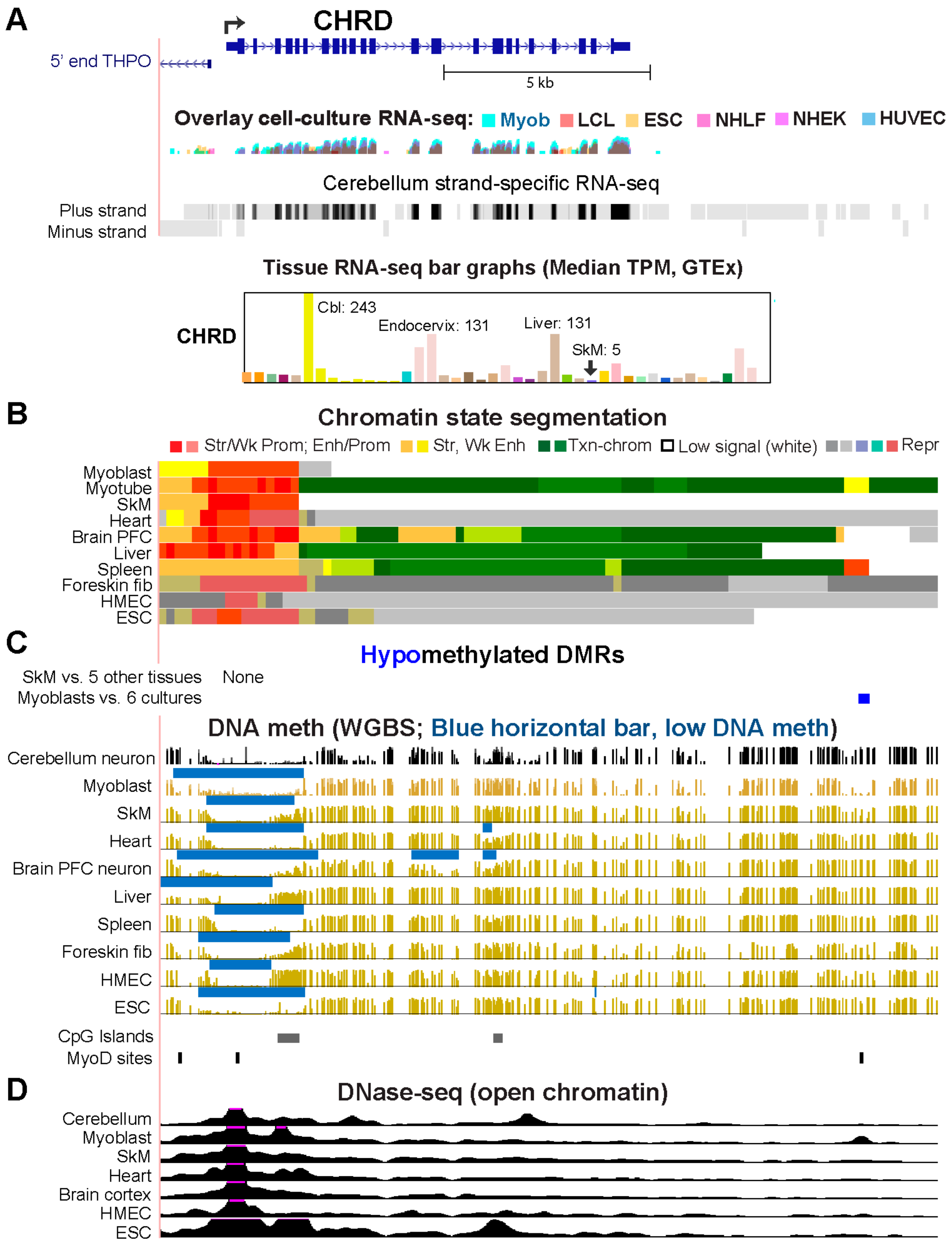

Figure S3. Intragenic hypomethylation in *CHRD* is associated with its upregulation in cerebellum. *CHRD* encodes a gene for chordin, a protein that is a bone morphogenetic protein antagonist implicated in various aspects of prenatal development [8]and expressed in neurons and astrocytes in adult rat brains [9]. The RefSeq Select isoform is shown at chr3:184,096,218-184,115,042. Panels **A-C** are similar to those described in Supplemental Figure S1. Panel **D** shows DNaseI hypersensitivity profiles from ENCODE/Duke University (vertical viewing range 0–0.2). Cbl, cerebellum and SkM, leg skeletal muscle in the GTEx bar graph.

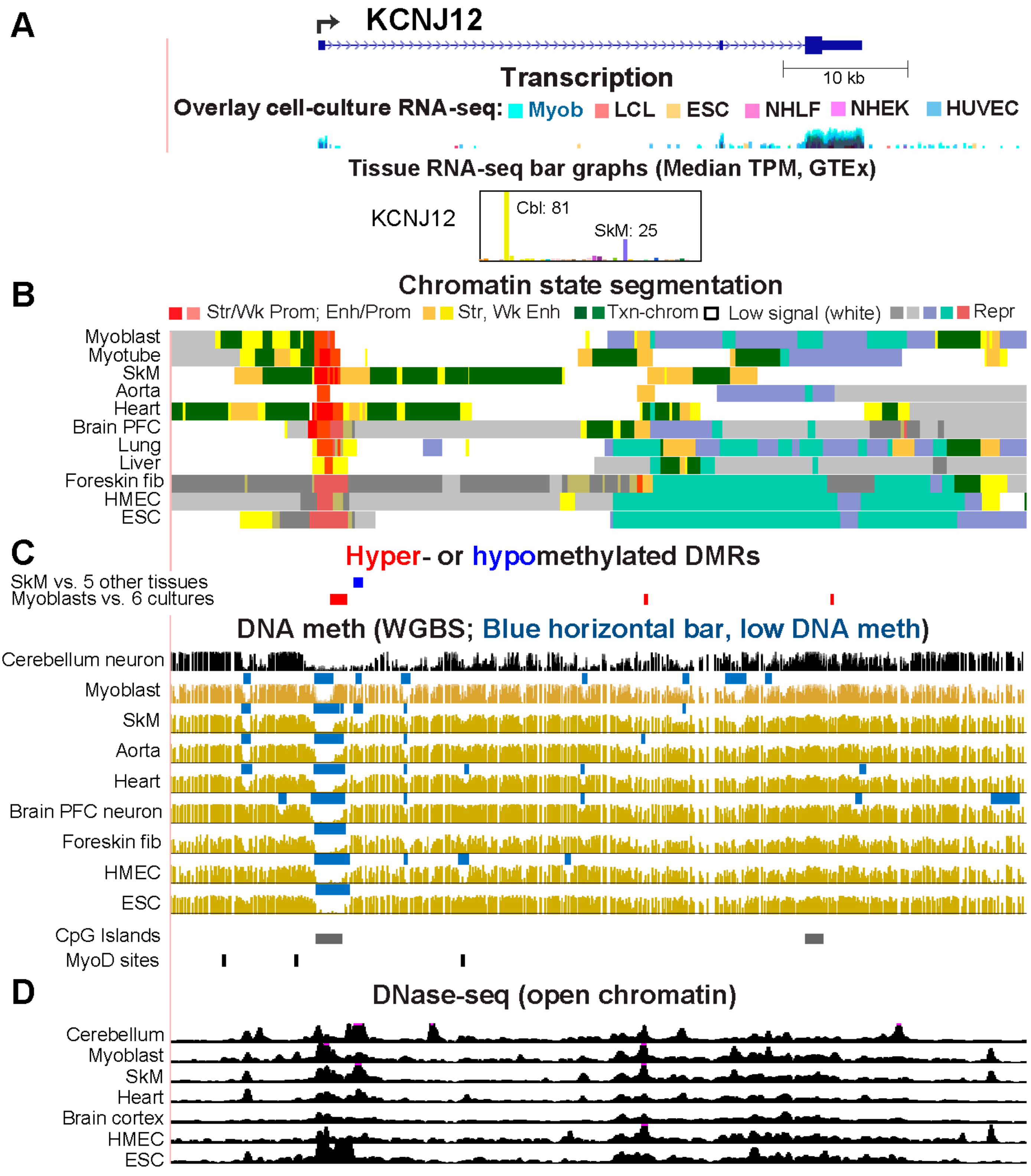

Figure S4. Cerebellar hypomethylation and myoblast hypermethylation in *KCNJ12* are associated with up- or down-regulation of this gene. *KCNJ12* encodes an inwardly rectifying potassium channel protein that may be involved in muscle regeneration after injury [10]. The single RefSeq Curated isoform shown is shown at chr17:21,267,797-21,336,264. Panels **A**-**D** are similar to those in previous figures.

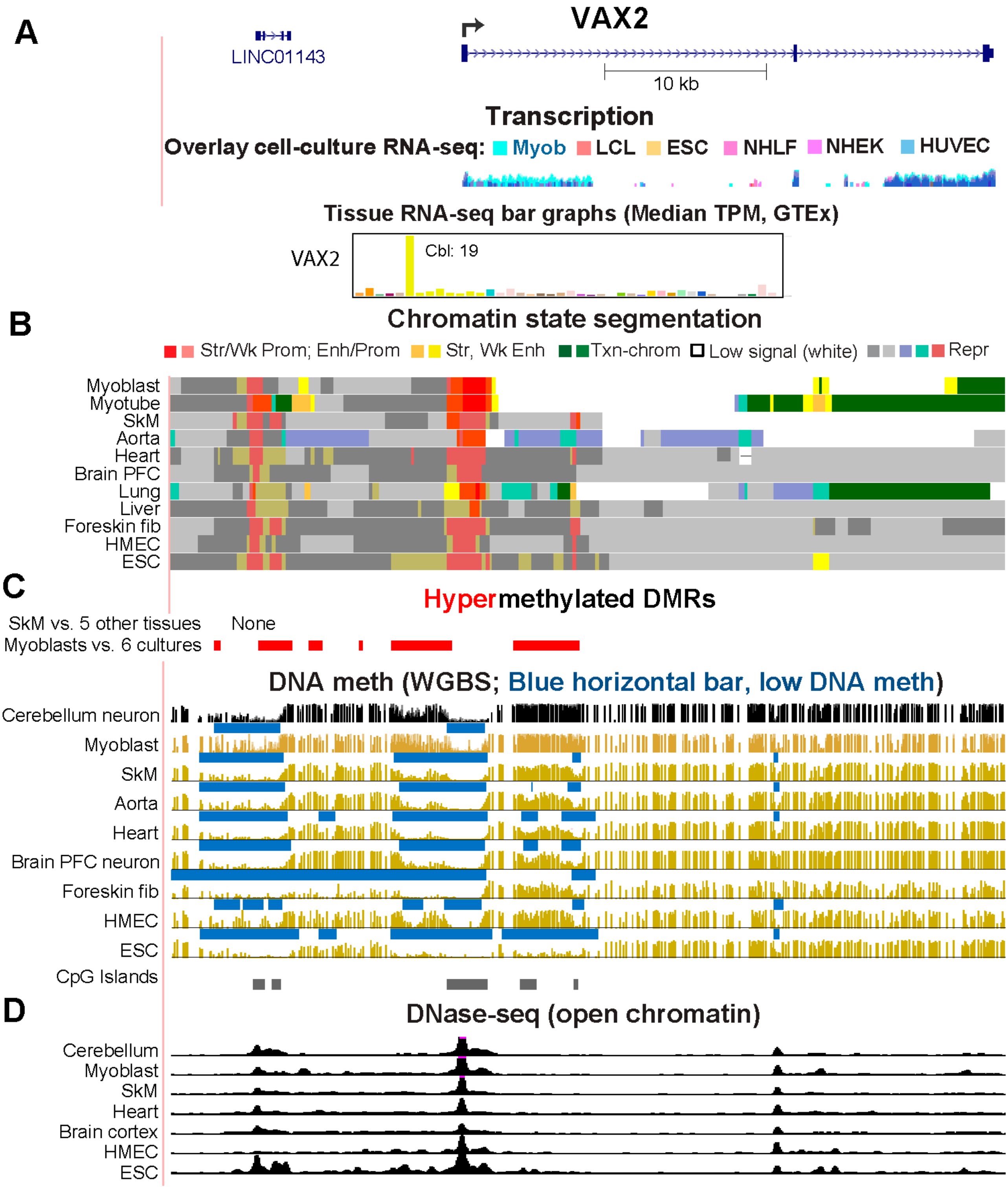

Figure S5. Hypermethylation upstream of the *VAX2* promoter in myoblasts and in intron 1 in myoblasts and cerebellum is associated with *VAX2* preferential expression in both cell populations. *VAX2* (ventral anterior homeobox 2) encodes a homeobox protein that is implicated in eye development [11] and may be involved in forebrain development [6]. The single RefSeq Curated isoform is shown at chr2:71,109,681-71,161,247. Panels **A-D** are similar to those in previous figures.

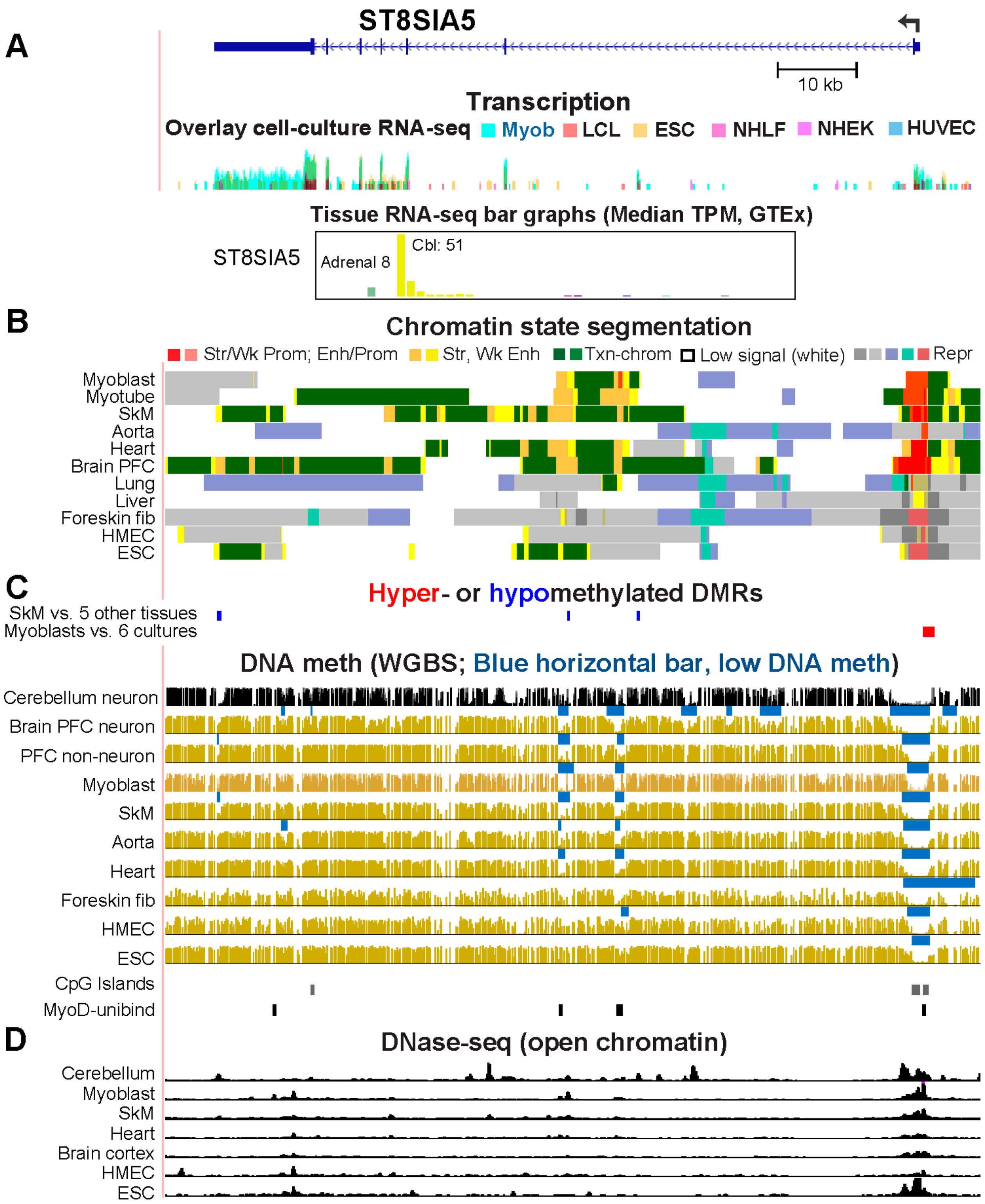

Figure S6. The promoter region of *ST8SIA5* in cerebellum is more extensively hypomethylated than that of brain prefrontal cortex. *ST8SIA5* encodes an α-N-acetyl-neuraminide α-2,8-sialyltransferase, a family of proteins important for brain development [12] and may play a role in myoblast differentiation [13]. The RefSeq Select isoform is shown at chr18:44,241,613-44,344,659. Panels **A**-**D** are similar to those in previous figures.

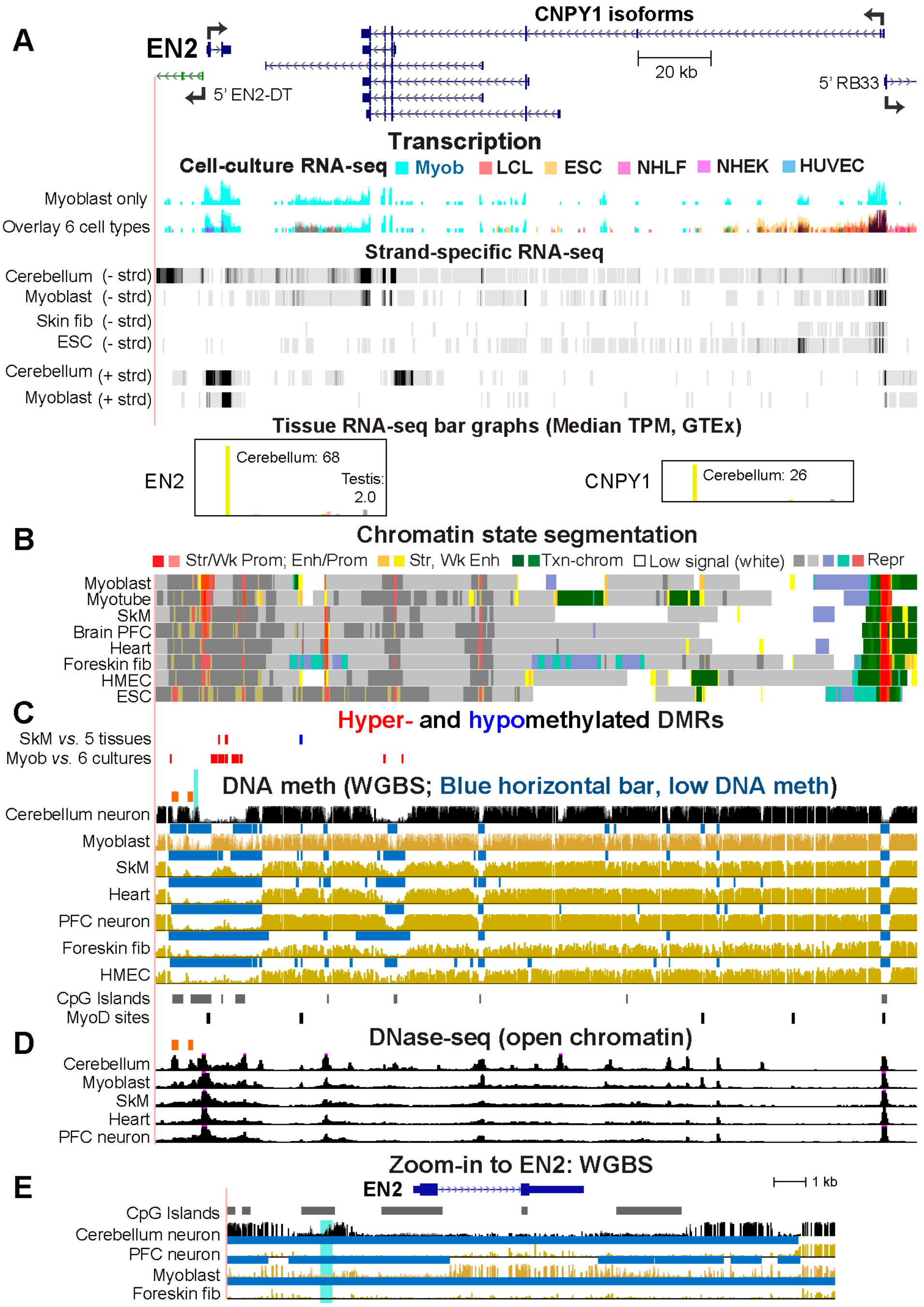

Figure S7. *EN2* and *CNPY1* have differential DNA methylation associated with tissue-specific expression levels and display cerebellar DNA hypermethylation in a promoter-upstream region previously associated with autism-linked hypermethylation. *EN2* encodes an engrailed homeobox protein while *CNPY1*, its downstream neighbor, encodes a signaling regulator. *EN2* is associated with cerebellar hypoplasia [14], postmitotic dopaminergic neuron development [15], and skeletal muscle fiber type specification [16]. The single *EN2* RefSeq Curated isoform and some of the *CNPY1* isoforms are shown at chr7:155,236,960-155,446,110. Panels **A-D** are similar to those in Figures S2 and S3. The cell culture RNA-seq tracks in Panel **A** shows both the overlay color-coded RNA-seq signal from six cell cultures and the individual profile for myoblasts. Panel **G** displays at higher magnification DNA methylation profiles for *EN2* and surrounding sequences. Blue-highlighting in Panels **C** and **E** indicates with blue highlighting the region shown to be significantly more methylated and hydroxymethylated in the cerebellum of autism patients than controls and to have higher *EN2* RNA and protein levels in patients [17,18]. The two nearby orange bars above the cerebellum tracks in Panels **C** and **D** indicate the position of DNase-seq peaks exhibiting low methylation surrounded by regions of high methylation uniquely seen in cerebellum.

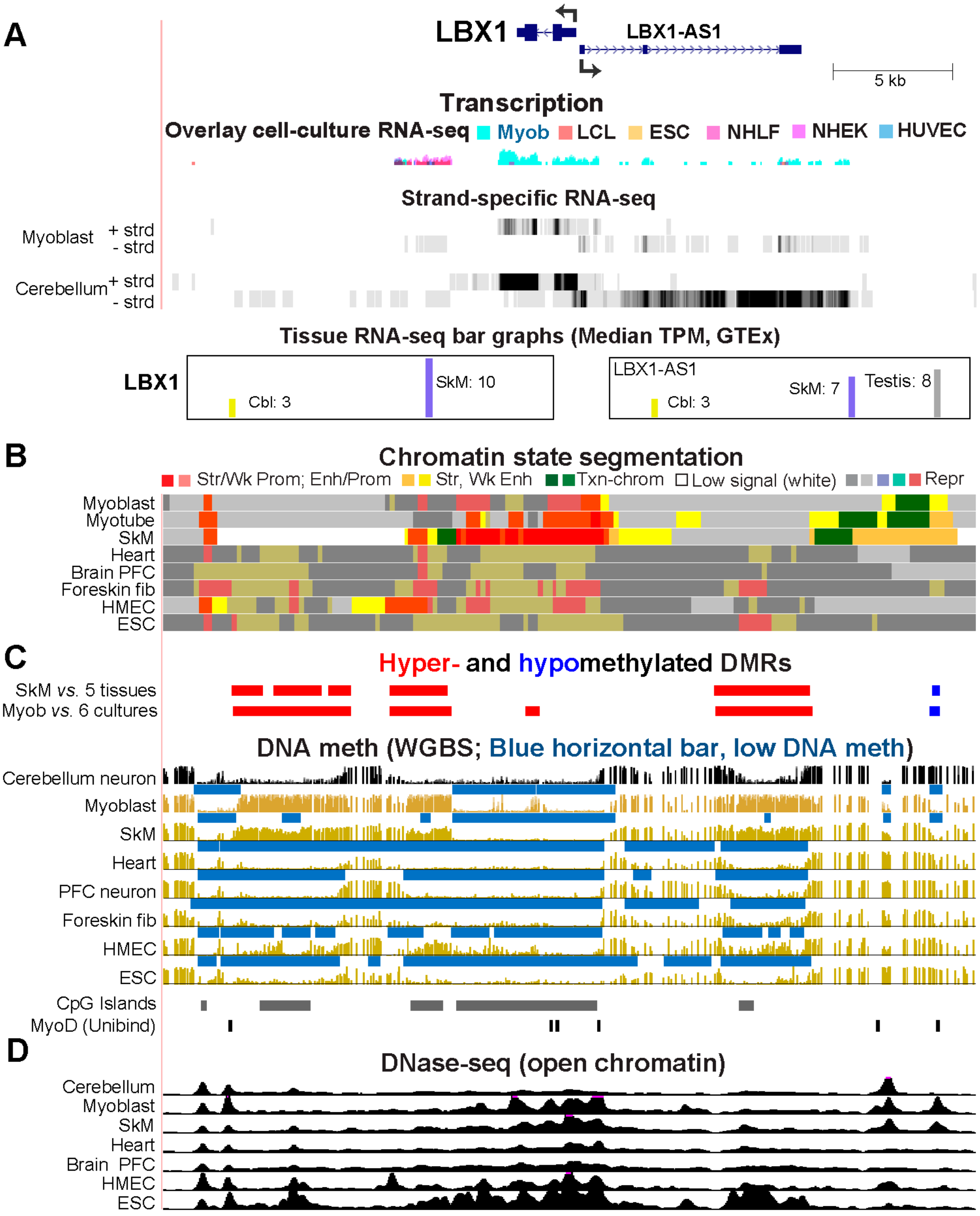

Figure S8. Extensive upstream hypermethylation in myoblasts and SkM and hypomethylation in cerebellum is associated with enhanced *LBX1* expression in muscle. *LBX1* encodes a homeobox gene required for development of neurons in the spinal cord and specifies pre-myogenic progenitor cells during embryonic SkM formation [19]. It is implicated in myogenic gene expression in myoblasts [20], and is necessary for normal SkM formation by acting downstream of *Pax3* in mice [21]. The single RefSeq Curated isoform is shown at chr10:102,971,927-103,005,906. Panels are similar to those in previous figures.

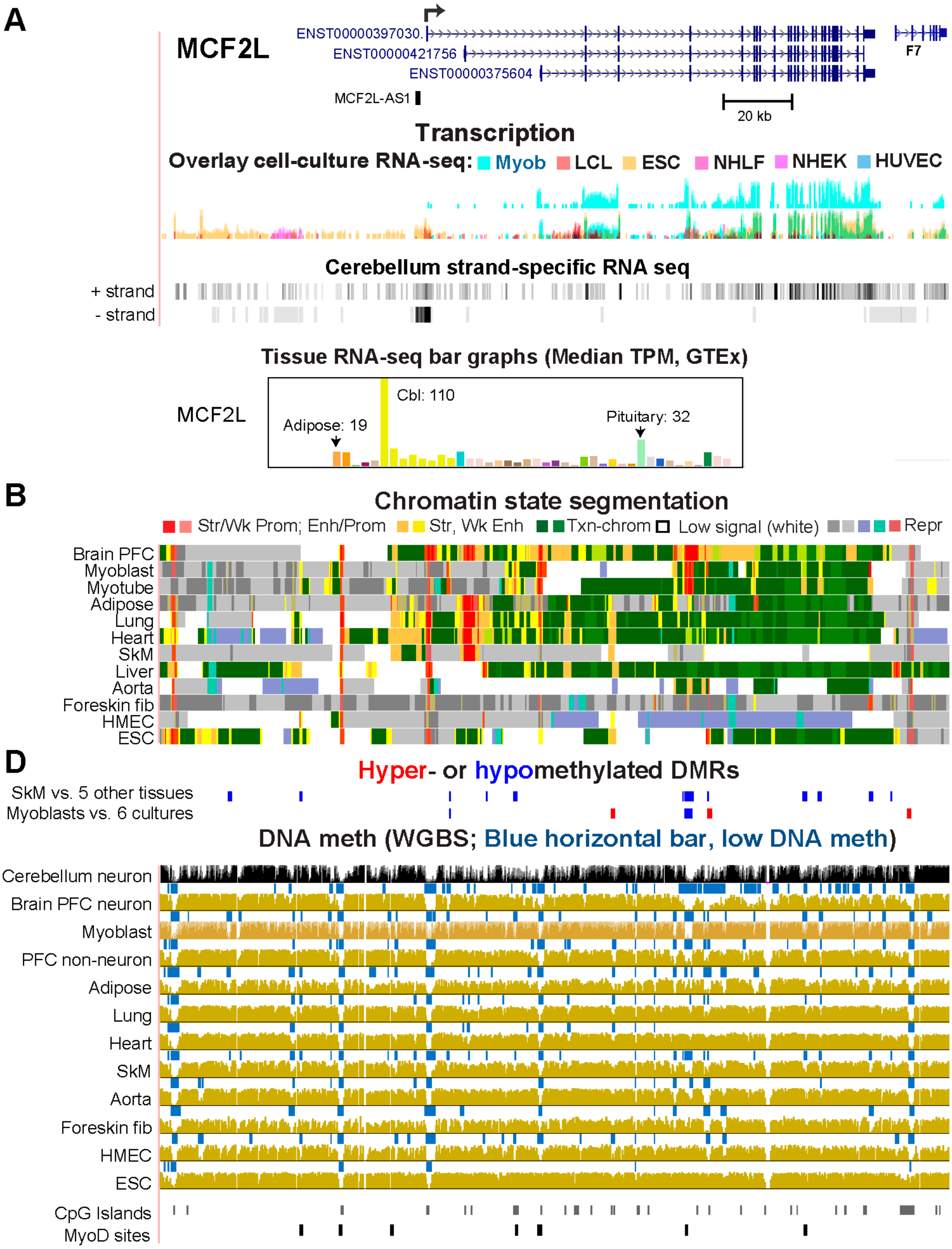

Figure S9. Myoblast DMRs in *MCF2L* likely affect promoter usage in cerebellum and myoblasts. *MCF2L* encodes guanine nucleotide exchange factor that has been mostly studied for its role and that of *MCF2L-AS1* in oncogenesis [6,22]. The region shown is chr13:113,544,791-113,775,670. As in Figure S7, Panel **A** shows both the individual strand-specific RNA-seq track for myoblasts and the overlaid non-strand specific signal RNA-seq profiles (two tracks; upper track is just myoblast profile; lower track is the overlaid signal from the indicated six cell cultures). Other panels are similar to those in previous figures.

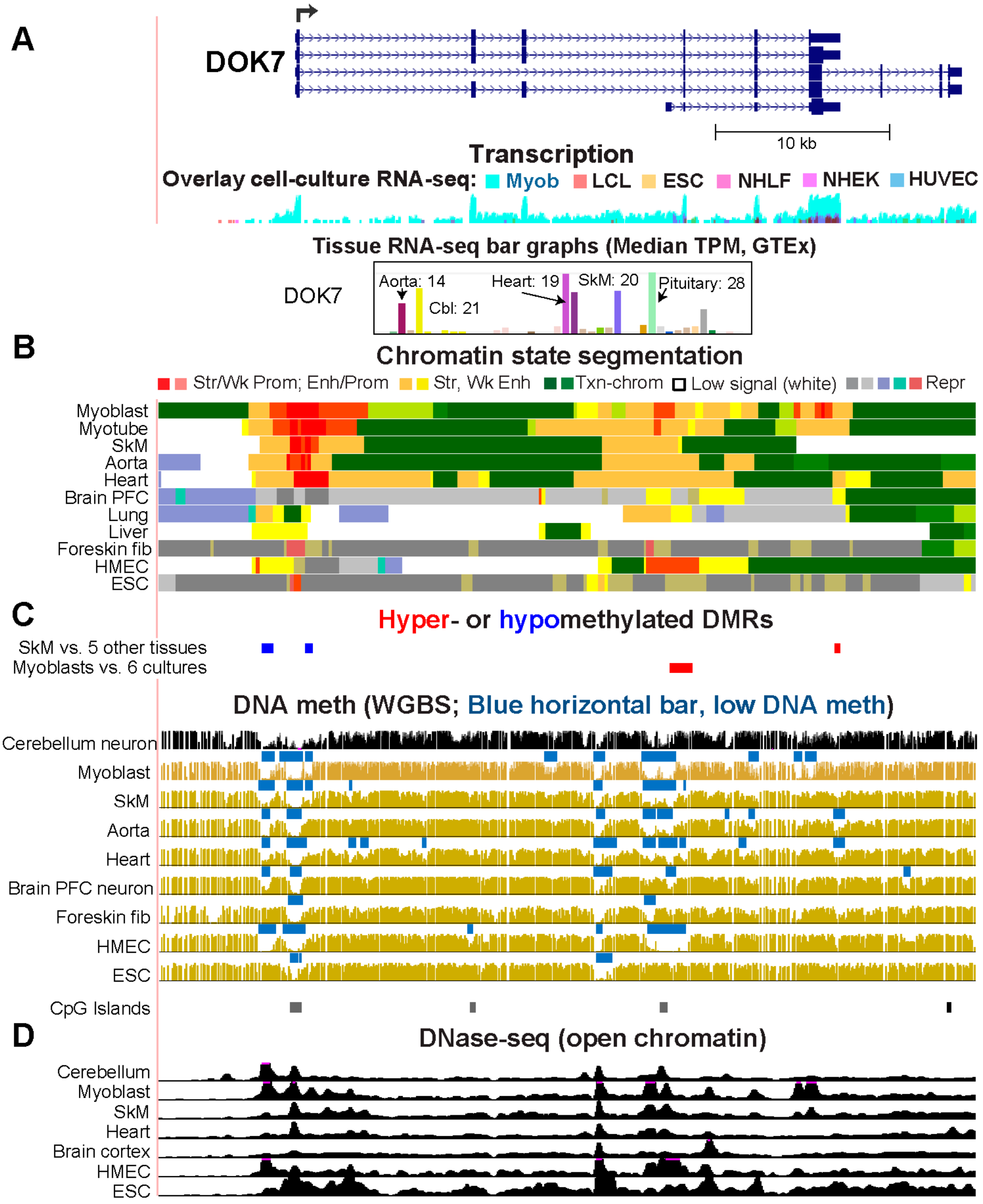

Figure S10. An intragenic region of myoblast and cerebellum hypermethylation is associated with repression of alternative promoter usage for *DOK7* in these cell populations. *DOK7* encodes a docking protein and is essential for neuromuscular synaptogenesis [23]. The RefSeq Select isoform is shown at chr4:3,457,238-3,504,003. Panels are similar to those in previous figures. Strand-specific RNA-seq profiles indicated that HMEC, unlike myoblasts and cerebellum, predominantly uses the alternative downstream promoter (data not shown).

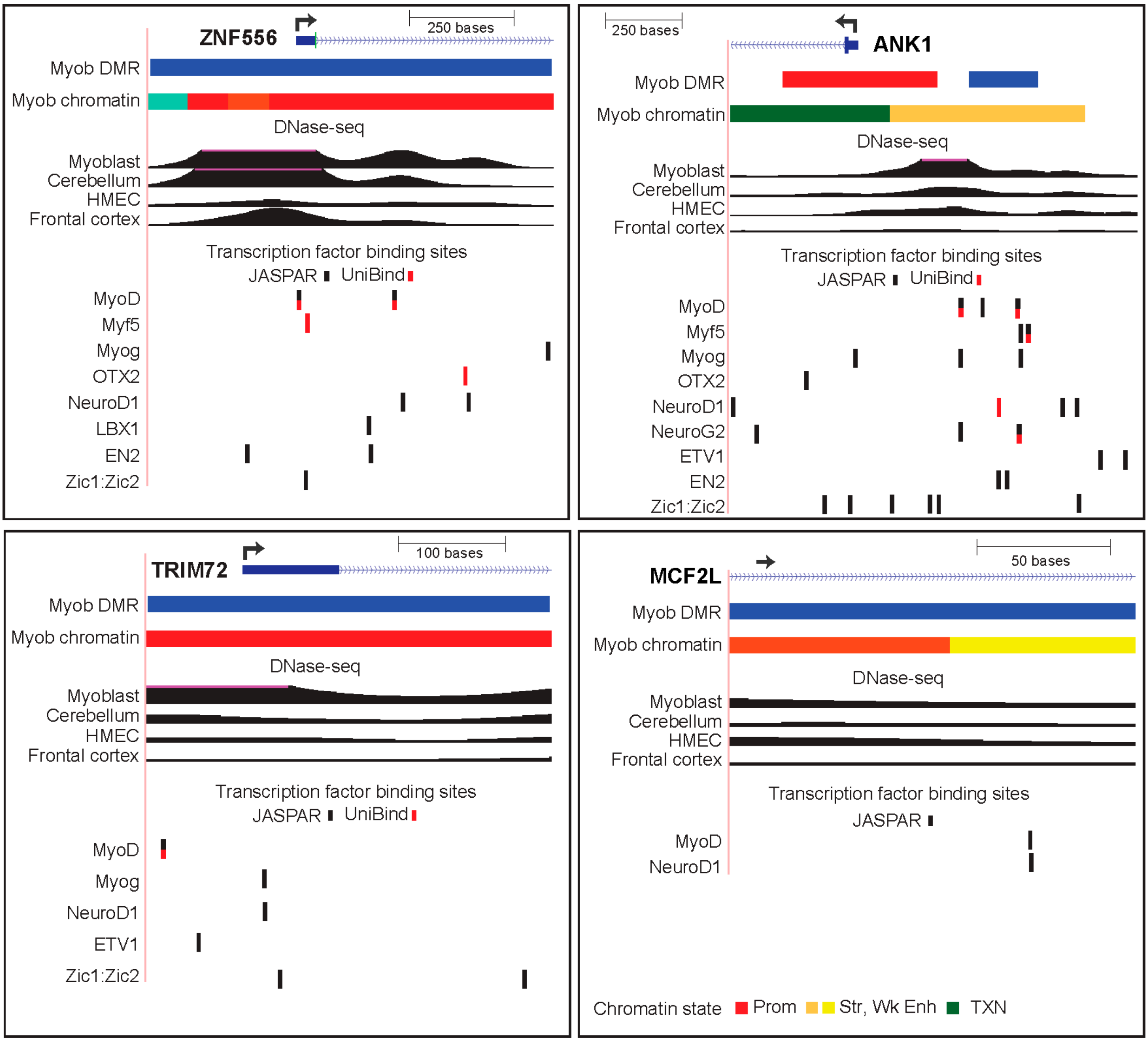

Figure S11. Transcription factor (TF) binding at four Myob/Cbl gene regions showing DNA hypomethylation in both myoblasts and cerebellum. Coordinates (hg19) for the regions shown are: *ZNF556*, chr19:2,866,622-2,868,563; *MCF2L*, chr13:113,629,238-113,629,542 (6 kb downstream of the most distal *MCF2L* TSS for RefSeq Curated isoforms); *ANK1*, chr8:41,654,321-41,656,977; *TRIM72*, chr16:31,225,264-31,226,003. Predicted sites for myoblast or brain-specific TFs were determined by JASPAR 2022 CORE with the minimum score set at 400. UniBind TF binding sites for myoblast or brain-related TF that were identified by ChIP-seq are as follows: MyoD sites in either myoblasts or a rhabdomyosarcoma cell line; NeuroD1 sites in medulloblastoma; NeuroG2 sites in fetal lung fibroblasts reprogrammed to neurons GEO Accession viewer (nih.gov); and OTX2 sites in a medulloblastoma cell line. The Myob hypomethylated region is shown as a blue bar above the Myob chromatin segmentation profile.

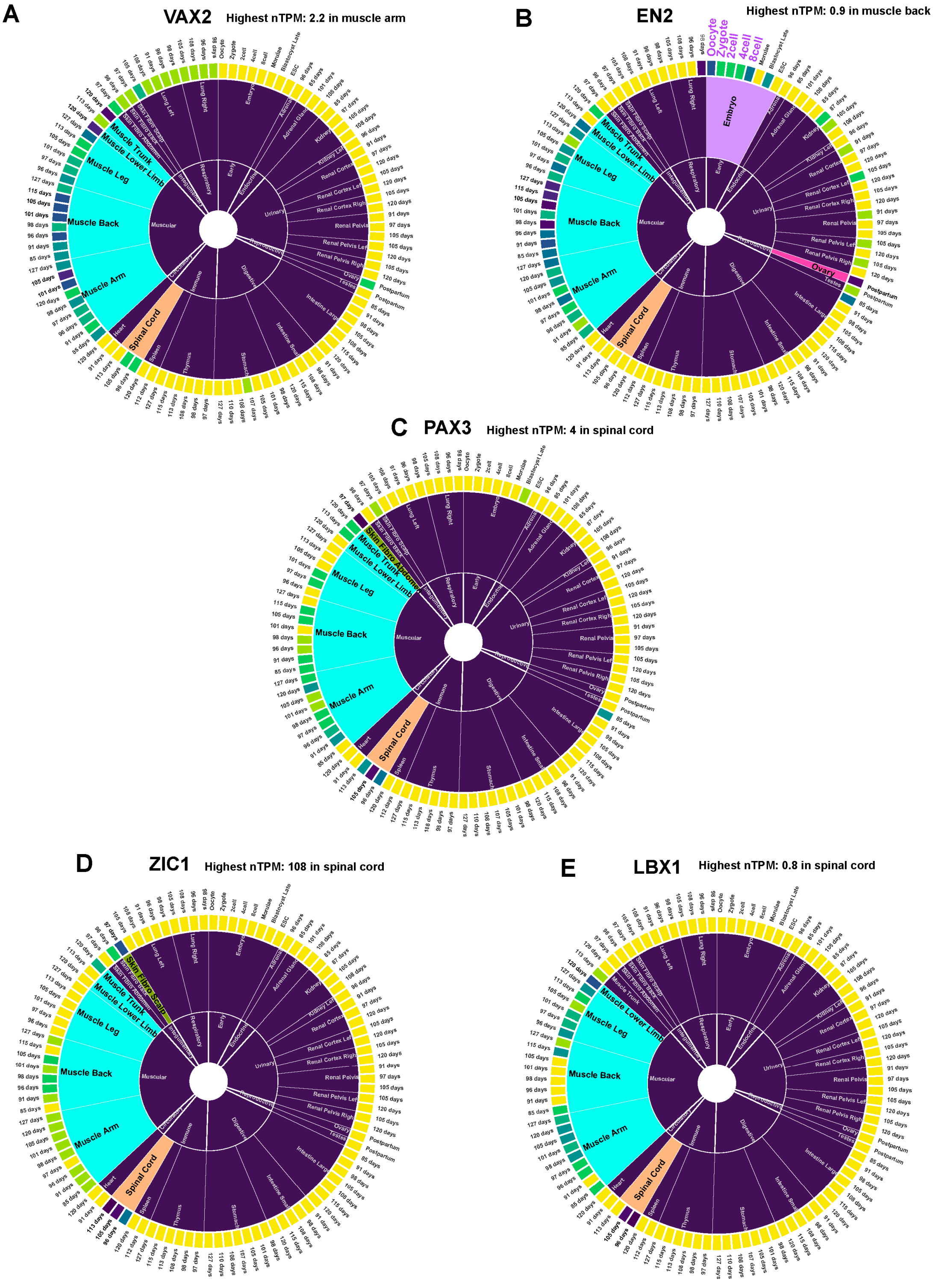

Figure S12 Relative mRNA levels for five of the Myob/Cbl genes in embryonic tissues or pre-implantation embryos. The relative mRNA levels are shown by the intensity of color in the outer bar in the pie charts with yellow indicating no detectable RNA (EmAtlas. Spatiotemporal Expression Analysis, Expression Analysis (imu.edu.cn)). Colored sectors highlight cell populations or tissues of interest. Relative RNA levels, which are in nTPM, are not scaled; the highest level for each gene is indicated in the heading for each panel. See Figure 4 for the analogous data for *ZNF556*.

References

1. Guo, Y.; Huang, C.; Xu, C.; Qiu, L.; Yang, F. Dysfunction of ZNF554 promotes ROS-induced apoptosis and autophagy in Fetal Growth Restriction via the p62-Keap1-Nrf2 pathway. *Placenta* **2023**, *143*, 34-44, doi:10.1016/j.placenta.2023.09.009.
2. Sirmaci, A.; Edwards, Y.J.; Akay, H.; Tekin, M. Challenges in whole exome sequencing: an example from hereditary deafness. *PLoS One* **2012**, *7*, e32000, doi:10.1371/journal.pone.0032000.
3. Aruga, J.; Shimoda, K.; Mikoshiba, K. A 5' segment of the mouse Zic1 gene contains a region specific enhancer for dorsal hindbrain and spinal cord. *Brain Res Mol Brain Res* **2000**, *78*, 15-25, doi:10.1016/s0169-328x(00)00057-7.
4. Aruga, J.; Millen, K.J. ZIC1 function in normal cerebellar development and human developmental pathology. *Adv Exp Med Biol* **2018**, *1046*, 249-268, doi:10.1007/978-981-10-7311-3_13.
5. Vandervore, L.V.; Schot, R.; Hoogeboom, A.J.M.; Lincke, C.; de Coo, I.F.; Lequin, M.H.; Dremmen, M.; van Unen, L.M.A.; Saris, J.J.; Jansen, A.C.; et al. Mutated zinc finger protein of the cerebellum 1 leads to microcephaly, cortical malformation, callosal agenesis, cerebellar dysplasia, tethered cord and scoliosis. *Eur J Med Genet* **2018**, *61*, 783-789, doi:10.1016/j.ejmg.2018.10.018.
6. Stelzer, G.; Rosen, N.; Plaschkes, I.; Zimmerman, S.; Twik, M.; Fishilevich, S.; Stein, T.I.; Nudel, R.; Lieder, I.; Mazor, Y.; et al. The GeneCards Suite: From gene data mining to disease genome sequence analyses. *Curr. Protoc. Bioinformatics* **2016**, *54*, 1.30.31-31.30.33, doi:10.1002/cpbi.5.
7. Pan, H.; Gustafsson, M.K.; Aruga, J.; Tiedken, J.J.; Chen, J.C.; Emerson, C.P., Jr. A role for Zic1 and Zic2 in Myf5 regulation and somite myogenesis. *Dev Biol* **2011**, *351*, 120-127, doi:10.1016/j.ydbio.2010.12.037.
8. Correns, A.; Zimmermann, L.A.; Baldock, C.; Sengle, G. BMP antagonists in tissue development and disease. *Matrix Biol Plus* **2021**, *11*, 100071, doi:10.1016/j.mbplus.2021.100071.
9. Mikawa, S.; Sato, K. Chordin expression in the adult rat brain. *Neuroscience* **2014**, *258*, 16-33, doi:10.1016/j.neuroscience.2013.11.006.
10. Hibino, H.; Inanobe, A.; Furutani, K.; Murakami, S.; Findlay, I.; Kurachi, Y. Inwardly rectifying potassium channels: their structure, function, and physiological roles. *Physiol Rev* **2010**, *90*, 291-366, doi:10.1152/physrev.00021.2009.
11. Alfano, G.; Shah, A.Z.; Jeffery, G.; Bhattacharya, S.S. First insights into the expression of VAX2 in humans and its localization in the adult primate retina. *Exp Eye Res* **2016**, *148*, 24-29, doi:10.1016/j.exer.2016.05.008.
12. Schnaar, R.L.; Gerardy-Schahn, R.; Hildebrandt, H. Sialic acids in the brain: gangliosides and polysialic acid in nervous system development, stability, disease, and regeneration. *Physiol Rev* **2014**, *94*, 461-518, doi:10.1152/physrev.00033.2013.
13. Go, S.; Go, S.; Veillon, L.; Ciampa, M.G.; Mauri, L.; Sato, C.; Kitajima, K.; Prinetti, A.; Sonnino, S.; Inokuchi, J.I. Altered expression of ganglioside GM3 molecular species and a potential regulatory role during myoblast differentiation. *J Biol Chem* **2017**, *292*, 7040-7051, doi:10.1074/jbc.M116.771253.
14. Sarnat, H.B.; Benjamin, D.R.; Siebert, J.R.; Kletter, G.B.; Cheyette, S.R. Agenesis of the mesencephalon and metencephalon with cerebellar hypoplasia: putative mutation in the EN2 gene--report of 2 cases in early infancy. *Pediatr Dev Pathol* **2002**, *5*, 54-68, doi:10.1007/s10024-001-0103-5.
15. Wallén, A.; Perlmann, T. Transcriptional control of dopamine neuron development. *Ann N Y Acad Sci* **2003**, *991*, 48-60, doi:10.1111/j.1749-6632.2003.tb07462.x.
16. Degenhardt, K.; Sassoon, D.A. A role for Engrailed-2 in determination of skeletal muscle physiologic properties. *Dev Biol* **2001**, *231*, 175-189, doi:10.1006/dbio.2000.0131.
17. James, S.J.; Shpyleva, S.; Melnyk, S.; Pavliv, O.; Pogribny, I.P. Complex epigenetic regulation of engrailed-2 (EN-2) homeobox gene in the autism cerebellum. *Transl Psychiatry* **2013**, *3*, e232, doi:10.1038/tp.2013.8.
18. James, S.J.; Shpyleva, S.; Melnyk, S.; Pavliv, O.; Pogribny, I.P. Elevated 5-hydroxymethylcytosine in the Engrailed-2 (EN-2) promoter is associated with increased gene expression and decreased MeCP2 binding in autism cerebellum. *Transl Psychiatry* **2014**, *4*, e460, doi:10.1038/tp.2014.87.

19. Shih, H.P.; Gross, M.K.; Kioussi, C. Muscle development: forming the head and trunk muscles. *Acta Histochem* **2008**, *110*, 97-108, doi:10.1016/j.acthis.2007.08.004.
20. Sdek, P.; Oyama, K.; Angelis, E.; Chan, S.S.; Schenke-Layland, K.; MacLellan, W.R. Epigenetic regulation of myogenic gene expression by heterochromatin protein 1 alpha. *PLoS One* **2013**, *8*, e58319, doi:10.1371/journal.pone.0058319.
21. Buckingham, M.; Bajard, L.; Chang, T.; Daubas, P.; Hadchouel, J.; Meilhac, S.; Montarras, D.; Rocancourt, D.; Relaix, F. The formation of skeletal muscle: from somite to limb. *Journal of anatomy* **2003**, *202*, 59-68.
22. Zhu, Y.; Yang, L.; Wang, J.; Li, Y.; Chen, Y. SP1-induced lncRNA MCF2L-AS1 promotes cisplatin resistance in ovarian cancer by regulating IGF2BP1/IGF2/MEK/ERK axis. *J Gynecol Oncol* **2022**, *33*, e75, doi:10.3802/jgo.2022.33.e75.
23. Burden, S.J.; Huijbers, M.G.; Remedio, L. Fundamental Molecules and Mechanisms for Forming and Maintaining Neuromuscular Synapses. *Int J Mol Sci* **2018**, *19*, doi:10.3390/ijms19020490.
